# Supplementary figures and images for: Dipteran flight diversity is shaped by aerodynamic constraints, scaling, and evolutionary trade-offs
Source: PLoS Biol. 2026 Jul 9;24(7):e3003473. doi: 10.1371/journal.pbio.3003473 (PMC13349162; doi:10.1371/journal.pbio.3003473)

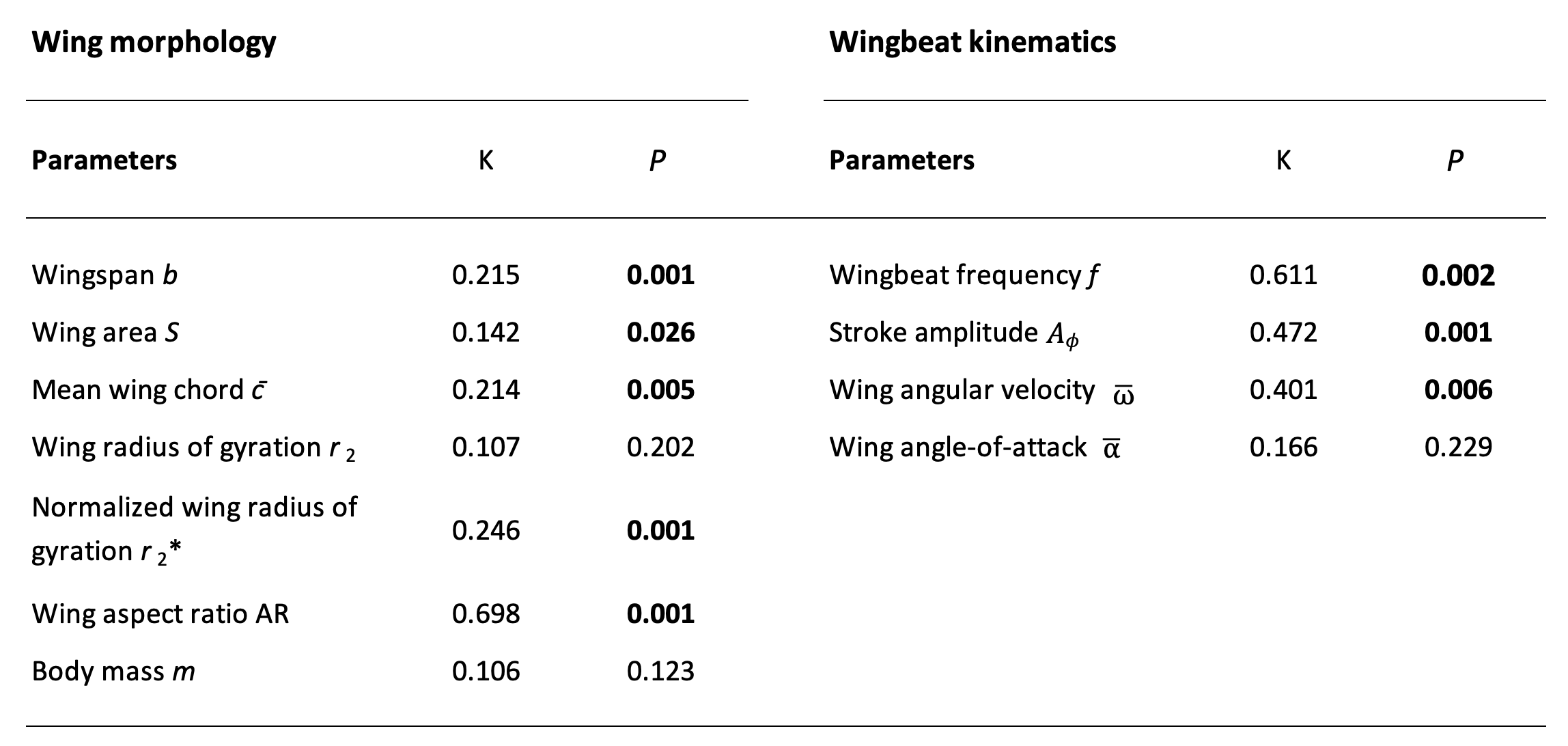

Supplement: S1 Table — Bold indicate significant p-values (p < 0.05). The data underlying this table are available at https://doi.org/10.5061/dryad.gxd25480s; see the Data Availability Statement for details. (PNG) [file pbio.3003473.s006.png]

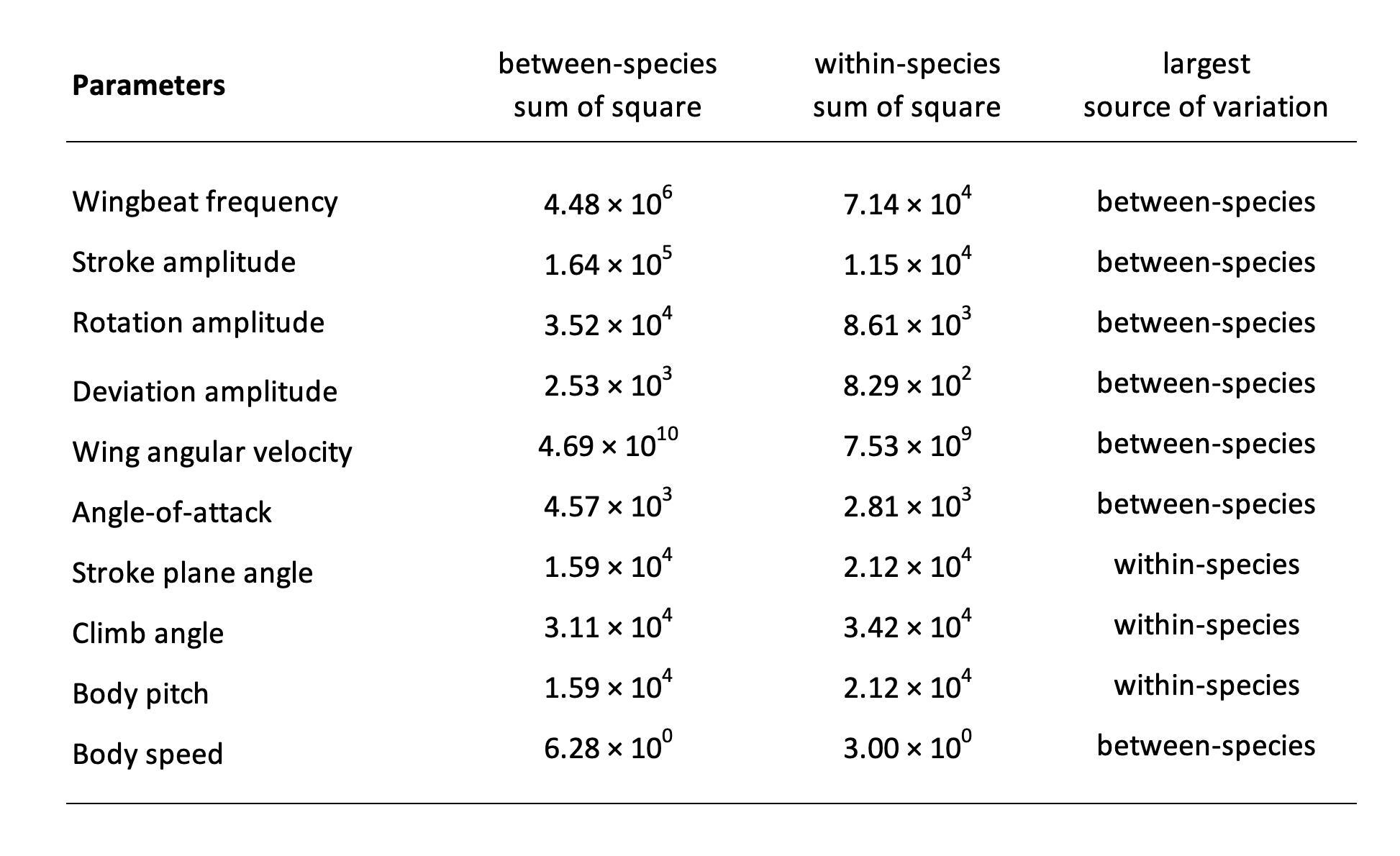

Supplement: S2 Table — The data underlying this table are available at https://doi.org/10.5061/dryad.gxd25480s; see the Data Availability Statement for details. (PNG) [file pbio.3003473.s007.png]
