# Supplementary material for: Dipteran flight diversity is shaped by aerodynamic constraints, scaling, and evolutionary trade-offs
Source: PLoS Biol. 2026 Jul 9;24(7):e3003473. doi: 10.1371/journal.pbio.3003473 (PMC13349162; doi:10.1371/journal.pbio.3003473)
Supplement: Attachment — Submitted filename: responses_to_reviewers_round1.pdf [file pbio.3003473.s010.pdf]

Reviewer #1:

Comment 1.1. This manuscript summarises a considerable amount of data. In this context it is certainly outstanding in its field, and will be of interest quite broadly, covering evolution, entomology, aerodynamics and acoustics.

Reply 1.1. We thank the reviewer for their positive evaluation and for emphasizing the broad interdisciplinary interest of our work, spanning evolution, entomology, aerodynamics, and acoustics.

Comment 1.2. I have three main issues:
1. The loss of exciting/ informative / important detail with the presentation (consider Figs 3 and 5D,E - lots of indistinguishable wiggly lines). I cannot currently assess the data availability. It would be nice to be able to compare and contrast any pair of measured insects from the PCs of the wing shapes and the Fourier terms for the kinematics. It should be pretty simple for me to recreate the SI movie (which is excellent) using measured - and reported - summarising statistics. Without this, (and I wouldn't want to go right back to early analysis) I am reliant on the story-telling as told, which I think misses much of the potential value of this study.

Reply 1.2. We agree that visualizing wingbeat kinematics for dozens of species in a single static figure can obscure detail. To address this, we now provide in our repository an interactive tool that enables phylogeny-aware filtering, subset visualization, and direct data export. In it users can select any family or arbitrary set of species and view only the corresponding kinematic traces and aerodynamic force profiles, thereby avoiding visual congestion. The tool also supports download of the exact numerical datasets used in this study, including wing morphology (shape outlines, key wing parameters), wingbeat kinematics (frequency, Fourier-series coefficients for the wing Euler angles $\phi(t), \eta(t), \theta(t)$), and time-resolved and wingbeat-averaged CFD-derived forces and power. These exports allow side-by-side comparison of any pair of measured insects and full reproduction of the figure-level summaries.

The tool is now referred to in the revised Discussion section and the Data Availability Statement (lines 750 and 1037, respectively). We appreciate the reviewer's constructive suggestion, which motivated us to build the interactive tool. This solution meaningfully improves interpretability for readers, and creates a durable, reusable resource for future comparative work in Diptera flight.

Comment 1.3. 2. The novelty and security of the take-home messages. Was it already known that craneflies were quite big and relatively slow-flapping? (Ellington certainly did some craneflies?). That robberflies had big muscles? That mosquitoes flapped with a high frequency, low amplitude, high incidence that didn't make sense in terms of aerodynamic power… but might (might) relate to sexual selection? That flappers with high aerodynamic power for their size (see 'efficiency' note below) also have high acoustic power for their size?

Reply 1.3. We agree that several individual observations have been reported previously; however, their comparative significance across Diptera has not been established. Our contribution is to place these facts in a unified, phylogenetically controlled framework across 46 species and a broad size range. For example, while mosquitoes are known to exhibit high aerodynamic and

acoustic power, our analysis shows that this elevation is chiefly relative to similarly small sized Diptera, whereas on an absolute scale they are not unusually high compared to the average Diptera. Likewise, patterns such as slow flapping in crane flies and enlarged flight musculature in robber flies are here quantified relative to appropriate size- and clade-matched baselines, rather than treated as isolated case studies. We have made these points explicit in the revised Introduction and Discussion sections (lines 155-162 and 738-749, respectively). We hope this clarifies that the novelty lies in the tested, size-aware, and phylogenetically informed comparative context, not in rediscovering known single-species attributes.

Comment 1.4. 3. The omission of some obvious mechanisms and sidestepping of analysis / logical flow.

Efficiency is not efficiency, or even 'aerodynamic efficiency' (per Rankine Froude). In the context of this paper this is a semantic/technical point. Useful power / Total power… in hovering, there is no useful power, so there is always zero efficiency. One aerodynamic efficiency applied to hovering considers the minimum power that would have been possible (for rotorcraft, this relates to disc area, weight, density etc.) as being 'useful'. The appropriate swept area is not obvious for flapping, and isn't really of interest. So I am happy for W/N to be used for the metric for economy (but I am not surprised that this would scale with size). A species with a high aerodynamic power demand for hovering may reasonably be viewed as being less 'economical' (I avoid the term 'efficient' for history above, but will cope if necessary) than a species of the same mass that has a lower aerodynamic power (for hovering). There is a spread of these economies (Fig. 7a). But note that - without the distraction of the lines - the spread is approximately even across scales, and there is a negligible relationship with mass (especially noting semi-log scale). So the focus here is on the relatively high power of the mosquitoes - reasonably related to their small amplitudes, high angles; but the same question arises at the larger end. Presumably the high-power demand large insects have relatively short wings - and this relates to induced power (see below).

The scaling discussion may end up invoking more interacting terms than can be handled simply here. Most notable is the omission of induced power. More later.

Reply 1.4. We agree that "efficiency" is not the appropriate term in the context of hovering, where useful mechanical output is not defined as a ratio. We therefore have removed this terminology throughout the revised manuscript, and instead refer now to "aerodynamic power" and "aerodynamic cost". Also, we explicitly state the scope and limitations of these quantities for hovering flight (see Methods, lines 339-341; Discussion, line 980-998).

Regarding the scaling analysis, our conclusions rest on phylogenetically controlled statistics rather than on visual trend lines. Using PGLS, we find that weight-normalized aerodynamic power (P_{aero}*) scales significantly with body mass (slope and 95% CI reported in the Results and Table 1), contrary to the impression that this relationship is negligible. The phylogenetic model is essential here because it accounts for shared ancestry and reduces undue leverage from clade-level outliers such as Culicomorpha (mosquitoes and midges).

On rereading the manuscript, we recognized that this statistical aspect was not presented sufficiently clearly. We have therefore revised the text to (i) state the PGLS framework explicitly,

(ii) report slope, 95% confidence intervals, intercept, and phylogenetic signal alongside each scaling claim, and (iii) add the PGLS result for P^*_{aero} versus body mass to Table 1. These clarifications are now included in the revised Methods section (lines 271-275); the corresponding new data are reported in the revised Results section and in Table 1. Moreover, in the revised figure 5, we now only show the PGLS fits and the theoretical slope based on our aerodynamic model, as we agree with the reviewer that the original 'distracting lines' are superfluent.

Finally, this is a comparative study aimed at macroevolutionary patterns rather than detailed aerodynamic decomposition for individual species. We therefore anchor interpretation in general physical scaling principles from biology (length/surface/volume relations) and aerodynamics (Reynolds-number effects on force and drag coefficients), and then test how Diptera depart from or conform to those expectations across the clade.

In the revised manuscript, we streamlined this rationale by adding a short validation note that links the CFD-derived forces and power to the scaling descriptors (Methods, lines 336). This note also clarifies which aerodynamic quantities the dataset can and cannot resolve. In addition, we highlight this in the dedicated "Scope and limitations" section of the Discussion (lines 954-998). These additions make explicit how the comparative framework is anchored in first-principles scaling and where its interpretative boundaries lie.

Comment 1.5. The propeller approach taken here is an adaptation of the quasi-steady blade-element analysis (see Ellington etc.)… but it entirely omits the induced power/drag aspect. To some extent this is fine as induced effects can be wrapped up into the D* metric (Fig 5) - but it does need to be mentioned if the high D* of the normal looking flies (sorry, cartoon looks a bit like a blowfly?) is going to be accounted for in comparison with the craneflies.

Reply 1.5. We appreciate this comment and agree that the distinction between modelling framework and interpretation should be made clearer. We do not use a quasi-steady blade-element analysis to estimate aerodynamic forces or power. Instead, we derive scaling relationships using Buckingham–Pi dimensional analysis to identify how forces and power must scale with morphology, kinematics, and fluid properties in flapping flight. As expected, this approach yields scaling relations that are similar in form to those obtained from quasi-steady blade-element arguments, but it does not assume quasi-steady aerodynamics or neglect unsteady effects.

Moreover, all aerodynamic forces and power reported in the manuscript are obtained directly from full-DNS CFD simulations, and therefore include induced effects, unsteady aerodynamics, wake interactions, and viscous contributions. In this context, induced power and induced drag are inherently captured in the resulting force coefficients and in the weight-normalized drag metric D^*.

Accordingly, differences in D^* between taxa, including the relatively high D^* observed in more "typical" flies compared to craneflies, already account for differences in induced and total aerodynamic costs. Craneflies require low power primarily because their low wingbeat frequency and large wings, leading to lower overall drag and induced power.

We now mention these aspects explicitly in our revised Methods, Results and Discussion sections (lines 303-321, 628-636 and 973-979, respectively). We hope that these clarifications resolves the reviewer's concern regarding the treatment and interpretation of induced aerodynamic effects.

Comment 1.6. Is viscosity the answer (Fig 5F)? And is it actually needed as an answer? It is not clear that there is an up-tick in aerodynamic power at small masses. The effect of viscosity would be fairly simple to determine by playing small and large insects through the CFD at high and low Re ('wrong') respectively - does the power shift as expected from the simplified viscosity account?

Note that D* around 1 is expected for a thin flat plate at 45degrees to the flow: without leading-edge suction, force will be approximately perpendicular to the surface. Alpha is not reported… does it relate to CD:CV throughout the flap?

Reply 1.6. We thank the reviewer for this constructive and technically important comment. Our viscosity-related scaling is based on well-established Reynolds-number dependencies that have been recognized since early fluid-mechanics research (Hoerner, 1965), and further quantified in the insect flight literature (Lyu et al., 2019). In our study, we use these Reynolds-number trends as physical benchmark expectations, not as assumptions, and explicitly test whether the CFD-derived aerodynamic performance of Diptera deviates from these expectations using phylogenetically controlled statistics.

Upon rereading the manuscript, we recognized that our rationale for the Reynolds-number analysis was not explained clearly and was not tested in a consistent way. We have therefore fully revised this part of our study. In the revised Introduction (lines 56-69), we now explicitly state that Diptera wings operate in the intermediate Reynolds-number regime, bounded by the high-Re inertial limit ($D^* \propto \mathrm{Re}^0$) and the low-Re laminar-viscous limit ($D^* \propto 1/\sqrt{\mathrm{Re}}$). Based on this framework, we replaced the previous Re-binning approach with a single PGLS applied to the full dataset. This removes arbitrary Re thresholds and treats Diptera as occupying an intermediate-Re continuum, bound by two asymptotic limits. The revised analysis is presented in the new Fig. 6 and in the updated Methods (lines 397-417), Results (lines 637-677), and Discussion sections (lines 871-893).

The reviewer is also correct that the relative drag metric D^* depends strongly on the angle-of-attack. For a thin flat plate in inviscid flow, the resultant aerodynamic force is expected to act nearly perpendicular to the wing surface; at an angle-of-attack of approximately 45°, this yields the baseline expectation $D^* \approx 1$. In the original manuscript we did not explicitly take this effect into account, which indeed limited interpretation of the observed variation in D^*.

In the revised manuscript we have therefore added a dedicated analysis to separate potential viscosity and angle-of-attack effects. This new analysis is presented in our newly introduced Fig. 6 and described in the Methods and Results sections (lines 397-417 and 637-677, respectively). In Fig. 6A we plot D^*against Reynolds number, and show with the corresponding PGLS regression that D^*scales significantly with Reynolds number. In Fig. 6B we relate D^* directly to the effective angle-of-attack, demonstrating that most species fall close to the geometric expectation based on angle-of-attack alone; in contrast, the smallest species exhibit

clear upward deviations from this pressure-based baseline, suggesting a Re-dependent viscous effect.

To confirm that these deviations arise from viscosity-driven tangential loading rather than from angle-of-attack effects, Fig. 6C shows the relative tangential (friction-dominated) force $T^* = C_T/C_N$ plotted against Reynolds number. T^* increases with decreasing Re and is markedly elevated in the smallest Diptera. This pattern matches the Re-dependent scaling expected for viscous drag and is supported by the PGLS regressions summarized in Table 1. Together, these results show that the elevated D^* in small Diptera is primarily caused by low-Reynolds-number viscous effects, not by differences in angle of attack.

We agree that additional CFD tests using deliberately mismatched Reynolds numbers could, in principle, further probe viscosity effects. In practice, however, systematic Reynolds-number sweeps of flapping wings have already been carried out in prior computational studies (Lyu et al., 2019), and our findings align closely with the Re-dependent behaviour described in those analyses. Given the comparative scope of the present study, we therefore interpret our results in the context of these established scaling patterns rather than adding another full set of Reynolds-manipulated CFD simulations.

We thank the reviewer for raising this point, as it prompted us to incorporate the new analysis presented in Fig. 6. This addition clarifies how angle-of-attack effects and low-Reynolds-number viscosity contribute separately to variations in D^*, and it strengthens the overall interpretation of our aerodynamic results. We hope these revisions satisfactorily address the reviewer's concerns regarding viscosity, angle of attack, and the interpretation of D^*.

Comment 1.7. The power calculations are couched in propeller terminology, with P* approx. Ud.D*. This approach entirely misses the possibility of reduced power demand through wake recapture: as can be seen from Fig 5D there are relatively high forces at the limits of downstroke/upstroke in the high frequency flappers, when the wing is moving slowest. This energetic benefit to wake recapture (see, for instance, some of Fritz Lehmann's work) would not be accounted for with the propeller analysis. I do not expect that mosquitoes will suddenly become low-power hoverers, but it is an obvious flaw with the approach. I expect the 'validation' referred to (L.397) might indicate a greater discrepancy between CFD and propeller reduction for the mosquitoes (CFD expected to calculate lower power due to the mechanisms above). A touch more on the validation would be comforting: even if it is only 'typical mosquito, typical Drosophila, typical cranefly, typical blowfly' to cover economical/uneconomical at both ends of the mass scale. Systematic error is not disastrous to the analysis, but it would be nice if it was small enough to be neglected.

Reply 1.7. We appreciate the reviewer's concern, but it made us realize that we should better describe our distinction between the CFD-based power estimate, and the model-based power scaling. All aerodynamic forces and power reported in our study are obtained directly from CFD. For example, we estimated instantaneous power from CFD as $\mathbf{P}(t) = \mathbf{T}_{\mathrm{aero}}(t)\cdot\mathbf{\Omega}(t)$, which we then averaged over the wingbeat cycle. The resulting total aerodynamic power thus inherently includes all unsteady aerodynamic mechanisms, including leading-edge vortex dynamics, wake capture near stroke reversal, and induced and viscous contributions.

The compact model relation $P^* \approx U_D \cdot D^*$ is used only as a dimensionally consistent surrogate to interpret interspecific trends in the comparative analysis; it is not used to compute the power values shown in the figures. We have now made this distinction explicit in the revised Methods section (lines 330-341), and we also clarify this point in the Results section where the surrogate is introduced and validated (lines 596-606).

Regarding wake recapture, we agree that this mechanism can reduce instantaneous aerodynamic power near stroke reversal, especially in high-frequency fliers. Because our aerodynamic power is computed directly from CFD, any energetic benefit of wake recapture is already reflected in the total P^*. We agree that this might not be captured by the surrogate $P^* \approx U_D \cdot D^*$. Therefore, as requested, we had a closer look at the validation of the surrogate with CFD (see Figure 1 below). This shows that the $P^* \approx U_D \cdot D^*$ model captures variations in power well, and that hereby the mosquitoes and midges are not strong outlier.

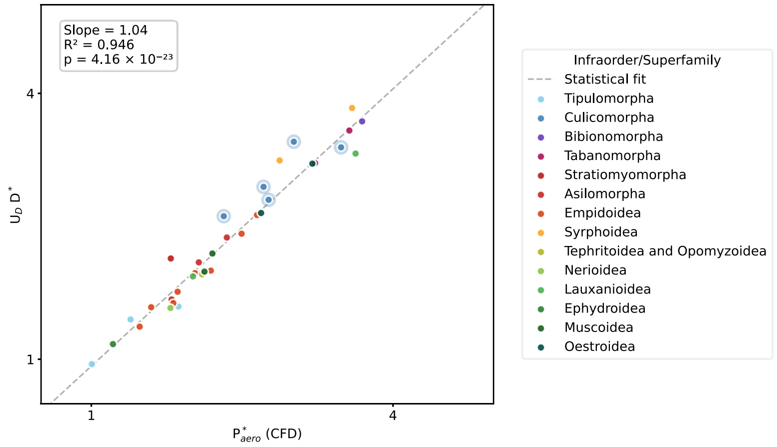

Figure 1. Comparison between CFD-derived aerodynamic power (P^*_{aero}) and model-based power ($P^* \approx U_D \cdot D^*$). Data for Culicomorpha are highlighted.

To keep the manuscript focused on comparative patterns rather than a detailed mechanism-by-mechanism aerodynamic decomposition for individual species, we do not expand the analysis further in this direction. However, all processed force and power time series are fully available for readers who wish to investigate such mechanisms in specific taxa. Also, the newly added interactive repository tool allows family-level filtering and direct export of the underlying data (see Discussion: lines 750-755; Data & Code Availability, lines 1037-1046).

We hope that these clarifications satisfactorily address the reviewer's concerns regarding wake capture and the use and interpretation of the $P^* \approx U_D \cdot D^*$ scaling surrogate.

Comment 1.8. How fair is it to infer that hovering with a relatively high aerodynamic power indicates low mechanical efficiency generally? 1) Other mechanical power demands may be considerable. Inertial power is worth a mention, even if it is then assumed that it can be neglected for some reason. It is one cost sometimes used to account for the general inefficiency of hovering in all sorts of animals. 2) Hovering does not represent all flight; it may be that an albatross hovers appallingly. Specialisation for sprinting, load-carrying, manoeuvring… would all demand compromise with hovering economy. While the sexual selection hypothesis for the peculiar mosquitoes is not unreasonable, others (particularly load carrying) are not precluded. While the measurements here are nice, they have not particularly shifted the debate.

Reply 1.8. We agree with the reviewer that these are important points, and we appreciate the opportunity to clarify the scope and interpretation of our analysis.

First, we fully agree that aerodynamic power does not represent the total mechanical power required for flight. Inertial power associated with accelerating and decelerating the wings can be substantial and is often invoked to explain the high energetic cost of hovering flight. In fact, we estimated inertial power based on the measured wing morphology and wingbeat kinematics. However, because including these estimates would substantially expand the scope of the manuscript and introduce additional assumptions regarding elastic energy storage and muscle–thorax coupling, we chose to focus the present study on aerodynamic power only. We now explicitly acknowledge this limitation and the role of inertial power in the Methods section (lines 340-342), and in the newly-added "Scope and limitations" Discussion section (lines 980-990).

Second, we agree that hovering performance does not necessarily reflect flight performance in other contexts. Many ecologically important flight behaviours in insects rely primarily on forward flight, including foraging, dispersal, migration, and predator–prey interactions. Specialization for these behaviours is therefore expected to involve trade-offs with hovering economy, such that elevated aerodynamic power requirements during hovering do not imply poor performance in other flight modes. That said, we chose hovering flight because it provides a well-defined and comparable reference condition across species, minimizing confounding effects of flight speed, trajectory, and behavioural context. We have now expanded the Introduction and Discussion to make this rationale explicit and to emphasize that hovering is used here as a standardized comparative benchmark, rather than as a proxy for overall flight performance (Introduction, lines 134-138; "Scope and limitations" Discussion, lines 987-990).

Finally, we agree that multiple selective pressures could contribute to the divergent flight characteristics observed in mosquitoes and midges, and that sexual selection is not the only possible explanation. Other factors, including load carrying (e.g., blood meals) or ecological demands on acceleration and manoeuvrability, are not precluded by our data. We have revised the Discussion to present sexual selection as a plausible and well-supported hypothesis rather than an exclusive explanation (lines 927-953); moreover, in the newly-added "Scope and limitations" Discussion section we extensively discuss the limitations of this hypothesis and acknowledge alternative selective pressures that may have shaped the observed patterns (lines 954-972).

We hope that these clarifications appropriately delimit the scope of our conclusions and address the reviewer's concerns regarding mechanical efficiency, flight mode specificity, and evolutionary interpretation.

Comment 1.9. Specifics.
I cannot agree that the data indicates a limit to small size: an unlabelled Figure 7 does not show higher aerodynamic power at the smallest size compared with the largest. A 'plateau in reduction' is pretty subjective. See above for ways of testing influence of viscosity.

Reply 1.9. We fully agree with the reviewer. Our data do not demonstrate a hard lower size limit, nor do they show that the smallest species require higher aerodynamic power than the largest. We

have therefore revised the manuscript to remove language implying a strict lower bound or a pronounced up-tick in power at small size. Instead, we now describe the observed pattern more cautiously as a diminishing reduction in weight-normalized aerodynamic power with decreasing size (see Discussion: lines 871-893). To avoid over-emphasizing this point, we also removed the small-size "limit/plateau" framing from the Abstract, the Results and Discussion sections (see Reply 1.6 for details). We hope that this properly address the reviewer's concern regarding the interpretation of a size limit.

Comment 1.10. I cannot agree that 'Scaling analysis show that smaller species compensate for reduced aerodynamic force production efficacy…'. A scaling analysis of hovering that does not include induced power is likely to be misleading. From memory, a Rankine-Froude derivation for induced power in hover would indicate an increased mass specific power demand with size (with isometric scaling of wing length) - as m^1/6. See Ellington 1984 (later papers).

But as above, I think the scaling analysis might best be avoided. There is enough data to compare different species of same mass.

Reply 1.10. We appreciate the reviewer's point and agree that a Rankine–Froude analysis of hovering predicts that mass-specific induced power increases with size when wing length scales isometrically, yielding the familiar $m^{1/6}$ trend (e.g. Ellington 1984). In the revised manuscript we explicitly reference this expectation and clarify how our framework relates to it (see Methods and Discussion, lines 337-339 and 868-873, respectively).

Our study uses two complementary components. First, all aerodynamic forces and powers reported in the figures and statistics are computed directly from CFD, which necessarily include induced effects and unsteady aerodynamics in full. Second, for comparative interpretation we employ a Buckingham–Pi-based scaling framework and a compact surrogate $P^{*} \sim U_D D^{*}$ that links morphology and kinematics to power in a dimensionally consistent way. Because weight support constrains U_D to scale approximately as $m^{1/6}$ under isometry and dynamic similarity, our surrogate recovers the same size trend that Rankine–Froude predicts for mass-specific power in hover. Deviations from this size trend can then be used to identify clade-specific adaptations or Reynolds-number-dependent variations in drag.

Regarding the suggestion to avoid such scaling analysis altogether, we respectfully prefer to retain it because it is central to the comparative evolutionary aims of the paper. The scaling framework places species-level CFD totals into a common, physics-based coordinate system and allows phylogenetically informed tests of whether Diptera conform to or deviate from morphological, kinematic, and dynamic similarity. We have tightened this presentation, added a brief validation note to clarify the role of the surrogate, and reduced extraneous text to keep the manuscript concise (see Methods: lines 330-341; Results, lines 596-606).

We hope that these clarifications make clear that induced effects are included in our power estimates, that our scaling interpretation is consistent with Rankine–Froude expectations at appropriate scales, and that the retained scaling analysis is necessary for robust comparative inference across the clade. Finally, to improve clarity, we also revised the wording in the Abstract and main text from "aerodynamic force production efficacy" to "force production capacity," to better reflect the physical meaning without implying a specific aerodynamic mechanism.

Comment 1.11. To conclude: lovely data, I trust it will become available in a useful form; scaling analysis problematic due to induced power (probably means intractable here); power calculations miss wake-recapture, but effect likely small (though would be nice to be quantified) and inertial power (to be acknowledged then dodged); uneconomical big flies need an account (I would point to induced power); viscosity argument to be validated; sexual selection argument for mosquitoes not significantly advanced (though interesting and worth considering).

Reply 1.11. We thank the reviewer for this thoughtful summary and for recognizing the value of the dataset. We largely agree with the assessment that this study necessarily prioritizes breadth over exhaustive mechanistic depth. Our goal is to provide a robust comparative framework across a large phylogenetic and size range of Diptera, rather than a complete decomposition of all aerodynamic and mechanical power components for individual species.

As clarified throughout the revised manuscript, all aerodynamic forces and power are computed directly from CFD and therefore include induced effects, unsteady mechanisms (including wake capture), and viscous contributions in an integrated manner. However, we agree that an explicit separation of induced power, wake-capture, and inertial power would require targeted analyses that go beyond the scope of a large comparative study. In the revised manuscript, we now explicitly acknowledge these limitations and outline them as directions for future work. To make this easy to locate for readers, we added for this a dedicated "Scope and limitations" section to the revised Discussion (line 954-998).

As explained in Reply 1.6, we have strengthened the treatment of viscosity by explicitly validating the Reynolds-number dependence of relative drag using angle-of-attack-based expectations and CFD-derived force coefficients. This shows that enhanced viscous effects at low Reynolds numbers drive the observed scaling of D^* (see the newly-added figure 6). We have also clarified the scaling framework and its relationship to classical induced-power arguments, and we now explicitly reference the Rankine–Froude expectation in the revised Methods and Discussion sections (lines 330-339 and 868-878, respectively).

Regarding mosquitoes and midges, we agree that sexual selection is unlikely to be the sole selective pressure shaping their flight characteristics. In the revised Discussion, we therefore present sexual selection as a plausible contributor (consistent with the observed aero-acoustic trade-off), while explicitly acknowledging alternative or complementary explanations such as load carrying (see Discussion, lines 927-972). We have also moderated language to avoid over-claiming where the data do not allow strong inference.

Finally, to ensure that the data are available in a genuinely useful form, we now provide full access to all processed morphological, kinematic, and aerodynamic data. Additionally, we now provide an interactive repository tool, allowing users to explore, filter, and export subsets of the data for more detailed analyses of specific taxa or mechanisms (see Discussion: lines 750-755; Data & Code Availability, lines 1037-1046).

We hope that these clarifications, validations, and explicit statements of scope adequately address the reviewer's concerns and that the revised manuscript strikes an appropriate balance between comparative insight and mechanistic detail.

Reviewer #2:

Comment 2.1. This paper examines the biomechanics and biophysics of flight in flies, integrating a variety of approaches. The paper is a methodological tour de force, ranging from phylogenetic comparative analysis, to flight kinematics, to geometric morphometrics, and acoustics. Moreover, nearly each analysis is rooted in first principles theory. I really appreciated the comparative approaches taken here, while most biomechanical studies focus on a single (or small number) of species. Here, we get a view across a megadiverse clade at what is similar and what is different across all this diversity, with insights to explain these patterns. I commend the authors on this detailed work, although I will admit I am not an expert on flight biophysics so hopefully other reviewers can judge the highly technical bits. I have some suggestions to improve the study.

Reply 2.1. We thank the reviewer for the thoughtful and encouraging assessment of our study and manuscript. Our aim was precisely to combine macro-evolutionary coverage with high-fidelity biomechanics, so we are pleased that the integration of phylogenetic comparative methods, geometric morphometrics, kinematics, CFD-based aerodynamics, and aero-acoustics came across clearly.

Comment 2.2. Comments:
There are a lot of analyses and results presented, and this comprehensiveness is a strength of the paper, but also can make it a bit hard to extract the key big picture conclusions. I don't have specific recommendations, but it would be nice to sharpen the key take-away points of general interest.

Reply 2.2. We agree that the breadth of analyses can make the main conclusions less immediately visible. In the revision, we sharpened the overarching messages by tightening the main text, moving secondary methodological details to the Supplement, shortened figure legends, and adding short signpost sentences in the Results and Discussion to highlight the key take-away points.

Comment 2.3. I found myself wondering how what we found is different than what we could have found. For example, could we have found that shape is highly conserved, but flies diversify their kinematics to have different flight abilities? Highlighting these also helps drive home the key points.

Reply 2.3. We thank the reviewer for raising this point. In our revised manuscript, we made the take home messages clearer by further developing the point suggested by the reviewer (i.e., the evolutionary lability of morphology vs. kinematics), in the Introduction (lines [134-162]), Methods (lines 373-396) and discussion section (lines 784-793).

Although more anchored in technical scaling hypothesis, our manuscript already provides alternative evolutionary scenarios regarding the coevolution of morphology and kinematics across size, through the single-metric weight-support scaling factors. Here, we derive the

expected exponents if weight support were maintained primarily by variation in only wing morphology or wingbeat kinematics, or a combination of both. Furthermore, we even test scenarios where weight support is maintained using only a single sub-metric, including morphological sub-metrics (wingspan *b*, r_2*, AR), wingbeat kinematics metrics (e.g., wingbeat frequency *f*, stroke amplitude A_ϕ), and even aerodynamics (e.g., C_F).

We have revised the text to make these alternative scenarios more explicit, both in the Methods section (lines 373-396), where we outline what we could have found under different evolutionary pathways, and in the Discussion (lines 784-793), where we contrast those expectations with the observed scaling patterns. This clarification strengthens the framing of what the study reveals about the evolutionary processes shaping Diptera flight diversity.

Comment 2.4. The differences between the mosquitos, craneflies, and the rest of flies is very intriguing. The authors attribute this to the need to use acoustic signaling for sexual selection (at least in part). Is it possible there was some key morphological-physiological innovation not visible in external wing shape that could have both allowed higher performance and at the same time constrained diversification within the larger radiation excluding these other lineages?

Reply 2.4. We appreciate the reviewer's encouragement to further explore possible evolutionary changes underlying the apparent canalization of wingbeat kinematics in more recently diverged lineages. Indeed, our study primarily focused on wing morphology, wingbeat kinematics, and aerodynamics, with more limited attention to muscle morphology. We agree that an unmeasured internal morphological or physiological traits, such as changes in flight muscle architecture, could have played a role in constraining flight evolution during the diversification of Diptera. To explicitly acknowledge this, we added a dedicated paragraph to our new "Scope and limitations" section in the revised Discussion (lines 991-998).

Comment 2.5. I was missing some broader context about how this study fits into what we know about insect and other animal flight. Are there lessons here that may generate hypotheses for other groups? For example, if we did this same study for Hymenoptera could we pose some hypotheses based on flies? Some forward looking comments will be welcome. I am not a flight or fly expert, but all the more so I am curious how this compares with the scaling other groups including mammal and bird flight and what universalities might exist.

Reply 2.5. We thank the reviewer for this relevant suggestion. We added a dedicated section to the revised Discussion, in which we place our results in a broader comparative context (lines 999-1020): "Broader comparative context and implications"). Here, we now highlight that large-size power limits in hovering are not unique to Diptera, but are expected across other insects, and in any flier operating in the intermediate Reynolds number regime. We also comment on how the conserved hovering kinematic template we identify in Diptera parallels similar constraints in other groups, whereas lineage-specific deviations (as in Culicomorpha) provide hypotheses for how ecological or sexual selection pressures might drive divergence in other taxa. These additions clarify how our findings extend beyond Diptera and may guide future comparative studies across insects in general, and even other flying animals.

Comment 2.6. L15 Basal- Calling species that are part of a more species-poor branch diverging early from the rest of the clade "basal" is a bit frowned upon now because it can be misleading

(along the lines of the fallacy that some species alive today are ancestors of other species). Do we have evidence that the ancestors at the base of the tree were more mosquito-like? This would require some knowledge of the fossil record (which I don't have for flies).

Reply 2.6. We appreciate this important expert input. There is no available evidence that the ancestors of Diptera were more mosquito-like. Therefore, to avoid such misinterpretation, we have (i) replaced "basal" with "earlier-diverged" throughout the revised manuscript, and (ii) added text to make explicit that present-day early-diverging lineages should not be interpreted as ancestral taxa (see Results and Discussion sections, lines 466-468, 777-779, respectively).

Comment 2.7.
L48: "evolutionary pressure forces". Maybe you mean, "evolutionary pressures"?
L68: resultS
L413 missing space
L492: modeS

Reply 2.7. Corrected.

Comment 2.8. L745 (paragraph): could this be interpreted as "many-to-one mapping" as is found in fish jaws, for example? i.e. there are many different morphologies that produce the same function. If so, it could help connect to other literatures.

Reply 2.8. We thank the reviewer for this insightful suggestion. We have added a bridging sentence in the Discussion (lines 768-770) noting that similar functional wingbeat outputs can arise from a wide range of wing and body morphologies. This pattern is consistent with a many-to-one mapping of form to function as described in other biomechanical systems, and we now cite Alfaro et al. (2005) and Wainwright (2007) to connect our findings to the broader ecomorphological literature.

Comment 2.9. 759: conflates "early branching" lineages with "early Diptera". It is not shown that the ancestral phenotype looked like mosquitoes or craneflies. Indeed Fig 2 seems to suggest their wings did not.

Reply 2.9. In connection with our response to Comment 2.6, we have revised the text to avoid any conflation between early-diverging extant lineages and ancestral Diptera. We now explicitly state in the revised manuscript that early-diverged lineages should not be interpreted as ancestral phenotypes (lines 466-468, 777-779). The sentence on line 759 of the original manuscript has been corrected to avoid confusing early-diverged taxa with early/ancestral Diptera (now lines 806-809).

Comment 2.10. L800: How can morphology be not constrained within younger clades but constrained on larger phylogenetic scales, since the former are nested within the latter? Clarify.

Reply 2.10. We thank the reviewer for pointing at this inconsistency in our discussion. We now discuss this result differently, proposing that potentially distinct mechanisms among different taxa lead to the observed unified scaling trend at the order scale (lines 856-861).

Reviewer #3:

Comment 3.1. In this comprehensive paper, Le Roy et al. explore the relationship between wing morphology, flight ability, and body size across a diversity of dipterans. First, they captured body size and wing morphology (size and shape) for 133 species. They then used a high-speed camera to determine the flight kinematics (e.g., wingbeat frequency, angle of attack, amplitude) of a subset of 46 species. Next, they used the morphological and kinematic data to model flight using Computational Fluid Dynamics, generating aerodynamic parameters such as lift and drag forces and aerodynamic power. Using these parameters, they explored how wing morphology, kinematics, and aerodynamics scale with body size and co-vary across phylogeny. Finally, they integrated the data to explore compensation mechanisms in small species and a possible trade-off between flight and acoustic power in mosquitoes. The amount of data collected is hugely impressive, and the data processing and analysis are well described (albeit with some caveats described below). I really enjoyed this paper and think it will be of interest to anyone interested in the evolution of insect wings, flight biomechanics, and the scaling of functionally well-elucidated morphology.

That being said, I have a number of comments that the authors should address.

Reply 3.1. We appreciate the positive feedback on our study and are pleased that the reviewer enjoyed reading the manuscript. We also welcome the acknowledgment of the broad audience our work may interest.

Comment 3.2. 1. This is a very long paper (~20,000 words), and very detailed, which makes it more challenging to follow than necessary. The introduction could be trimmed down substantially, and most of the methods could be moved to the supplementary material. I do not work in flight biomechanics, and while I could follow the methodology, at times it was difficult to see the wood for the trees—that is, the details obscured the hypotheses being tested. It would be clearer to focus on which parameters are assessed at each stage of analysis (geometric parameters from the morphological analysis, kinematic parameters from the high-speed camera, and dynamic parameters from CFD). The focus should then be on the biological meaning of these parameters and how they relate to the hypotheses, rather than on the method by which they were collected. This structure (morphological, kinematic, and dynamic) is laid out clearly in Figure 4, but not consistently elsewhere in the paper.

Reply 3.2. We thank Reviewer 3 for their feedback regarding the density of the manuscript. While we aimed to present the complexities of our analysis thoroughly, we recognize that the methodological detail may at times overshadow the primary biological hypotheses. We note that this feedback aligns with Comment 2.2 from Reviewer 2. Accordingly, we have taken steps to streamline the text.

In the revision, we sharpened the overarching messages by tightening the main text, moving secondary methodological details to the Supplement, shortened figure legends, and adding short signpost sentences in the Results and Discussion to highlight the key take-away points.

Although we streamlined the manuscript as much as we could, we have retained the core methodology within the main text. Because our study integrates distinct disciplines (evolutionary biology, comparative biomechanics, and aerodynamics), we believe it is essential for the reader to understand how these layers of data are interconnected. We chose PLOS Biology specifically because it allows for this comprehensive integration.

Comment 3.3. 2. More generally, the paper should be more firmly structured around specific hypotheses (e.g., the relationship between wings used for flight versus wings used for sound production; the impact of small body size on wing shape and function). The introduction explicitly positions the dataset as essential for understanding the selective pressures shaping insect flight and flight morphology, yet these selective pressures are not very clearly articulated. As written, the reader is somewhat overwhelmed—though also impressed—by the sheer quantity of data.

Reply 3.3. We agree with the reviewer that the large volume of data in this study requires a more explicit hypothesis-driven structure. We have now revised the Introduction (lines 134-154) to clearly articulate the three hypotheses (named “objectives”) we are addressing. Furthermore, we have added 'signposting' sentences to these objectives in the revised Results section (lines 432, 535, 680, 702), and we now connect our findings to the objectives more explicitly in the Discussion section (lines 757, 825, 931), ensuring the reader can more easily navigate the data.

Comment 3.4. 3. Figure 4. The PGLS was fit to log-transformed data (L480), yet the y-axes are sometimes log-transformed (e.g., Fig. 4A) and sometimes not (e.g., Fig. 4C). Further, based on Table 1, the PGLS was a single-predictor linear regression with a slope and intercept. How, then, does the PGLS in Figure 4D appear curved when plotted on a log-log scale? Similarly, if there is a linear relationship between the logs of x and y, why are the fits not curved when plotted on a log-linear scale (e.g., Fig. 4G)?

Reply 3.4. The results of the PGLS regressions reported in Table 1 were indeed obtained using log-transformed variables. However, in Figure 4, we chose to present the scaling of most traits using linear-log (semi-log) axes because a linear Y-axis is more physically intuitive for interpreting parameters like angles and coefficients. Furthermore, these traits (except for wingspan, which is shown in log-log) do not vary over a large range of values. We realize this choice was not explicitly explained in the original text; we have now edited the Figure 4 caption to be clear about this distinction.

Regarding the reviewer's question as to why the PGLS fit in Figure 4D appears curved: this is because Figure 4D is plotted on linear-log axes (linear Y-axis, log X-axis), not log-log axes.

Regarding Figure 4G, the expected fit is indeed a curve, but the changes in the force coefficient (named C_F in the revised manuscript) across body masses is very small (scaling factor = 0.037). The curve is technically curved, but it is so weak that it looks like a straight horizontal line. Similarly, for the other parameters that vary only slightly across the mass range, the predicted relationships are technically non-linear but appear linear due to the small magnitude of variation.

Comment 3.5. 4. Equation (11) relies on an assumption that is not fully addressed. The assumption is that as the observed scaling relationship diverges from the expected relationship under morphological/kinematic/dynamic similarity and moves toward the slope expected under weight support, the parameter Δa = (a_allo - a_sim) increases. Implicitly, the authors assume that the larger the Δa, the more the trait is adapted to meet the requirement of weight support. However, Δa will increase as the slope of the allometry moves away from that expected under similarity, regardless of whether it moves toward the weight-support slope (as seen in Fig. 4G). This should be accounted for. Relatedly, in Figure 4H, C_v has a negative %, which indicates that the slope of the observed allometry is less than expected under dynamic similarity, but it is not necessarily true that it is also moving away from the slope for weight support, as implied by the y-axis (although in this case, by happenstance, it is). Further, by focusing solely on the slope, Equation (11) ignores the intercept; one can imagine a scenario where the slope remains the same but the intercept changes, bringing the observed scaling relationship closer to the expected weight-support relationship. In short, there are hidden assumptions in Equation (11) that should be made explicit.

Reply 3.5. We thank the reviewer for this insightful comment, as they are correct that our previous formulation based on $\Delta a = a_{\text{allo}} - a_{\text{sim}}$ could increase even when a trait's slope moved away from the weight-support expectation, and thus did not reliably capture directional contributions. It also implicitly assumed that any deviation from similarity contributed positively, which was not always the case. To address this, we revised the manuscript to use the relative, directional allometry metric introduced by Le Roy et al. (2025), defined as

$$a^* = \frac{a_{\text{allo}} - a_{\text{sim}}}{a_{\text{ws}} - a_{\text{allo}}} \cdot 100\%.$$

See the revised Methods section for details (lines 391-396). This new formulation explicitly measures movement from similarity toward the single-trait weight-support slope, thereby resolving the ambiguity noted in Fig. 4H. Positive values now indicate movement toward weight support; negative values indicate the opposite. These revisions improve both clarity and interpretability of our data, and the updated values are now used throughout the manuscript and in Fig. 4H. The directional metric is now applied consistently throughout the text and Fig. 4H, replacing the earlier Δa formulation.

Comment 3.6. 5. Figure 5A. The authors should be explicit about which flies rely on acoustic signals for mate localization and which do not, and these should be indicated on the plot. Also, I think the lines may be mislabeled: the dot-dash line is labeled "low frequency fliers" but corresponds to species with higher wingbeat frequencies. It is also not clear how these lines were calculated. They are not fitted lines, but are instead derived from the model. In that case, how were the intercepts for the two groups calculated? The same issue applies to Figures 7A and 7B. In the caption for Figure 7, the authors state: "Trend lines are based on scaling laws (see Fig. 5) and a statistical fit." How was this statistical fit achieved?

Reply 3.6. We thank the reviewer for helping us to improve these figures. We revised the figures and accompanying text as following:

- We now explicitly identify the species that use acoustic signals for mate localization (Culicomorpha) using a thick black outline in the Figures 5 and 7.
- In the revised Methods section, we now clarify how the model-derived expectation lines were generated (267-275, 397-417). The revised figures now show only two types of lines: the PGLS fits and the predictions from our dimensional scaling models.
- The lines in Figure 5A were indeed mislabelled; these lines have been removed in the revised manuscript, following the changes described in our replies to Comments 1.2.

Comment 3.7. 6. In general, the authors overstate the causal implications of their results. While the data are consistent with aerodynamic constraints in general and weight support specifically, and with potential trade-offs involving acoustic production, they are essentially correlational. Future analyses that incorporate phylogenetically independent contrasts would be helpful in establishing causality, but are outside the scope of the current paper.

Reply 3.7. We agree that our conclusions regarding potential trade-offs between aerodynamic cost and acoustic production are primarily correlational. In the revised manuscript, we have therefore toned down this statement (lines 927-953). Moreover, we now explicitly note this limitation in the new "Scope and limitations" section of the Discussion, where we also mention the future analyses suggested by the reviewer (lines 955-965).

Comment 3.8. 7. L. 559 and L188. The authors measured body length and width. A little more detail here: length is presumably tip of head to the terminila? But how is thorax width measured? On what plane? An image showing example measurements as a supplemental figure would be very helpful.

Reply 3.8. As requested, we added the requested details on the anatomical definition and measurement plane for body length and thorax width in Supplementary Methods S1.2.

Comment 3.9. 8. A minor point, but the terminology surrounding scaling, allometry, isometry, and similarity is used somewhat inconsistently. For example, isometry has a very specific meaning (literally no change in proportion), yet here it is used more liberally to mean scaling that conforms to expectations under geometric similarity (Table 1). The term similarity is also used in a way that sometimes obscures meaning. Although it is defined at L456, the authors switch between morphological similarity and isometric scaling (L460), giving the impression that similarity equates to isometry, which is not always the case. Perhaps "proportionate" versus "disproportionate" scaling would be clearer. This is admittedly a bit of a minefield, but the authors should think carefully about vocabulary to maximize clarity.

Reply 3.9. As requested, we revised our terminology throughout the revised manuscript and the Supplement. We removed all informal uses of "isometry" and now consistently refer to "geometric similarity" when describing expected proportional scaling. We thank the reviewer for helping us improve clarity and avoid confusion between isometry and similarity.

Reviewer #4:

Comment 4.1. This is a short review, necessarily so because of time constraints, but also because the paper is very good and requires little comment

This is a very impressive work, collecting the specimens, the morphological analysis, flight and kinematics analysis, CFD - the authors have really pushed the limits. The paper is well written, dense, but accessible. The results are presented clearly. If nothing else, the collection of data and the scaling is fascinating. I particularly appreciate the "simple" models that are used to tie the quantitative and descriptive components of the analysis together.

Reply 4.1. We thank the reviewer for the very positive evaluation of our work. We appreciate the acknowledgment of the breadth of the dataset, the integration of morphological, kinematic, aerodynamic, and modelling approaches, and the clarity of the presentation. We are grateful for this encouraging feedback.

Comment 4.2. I don't have anything major to object to but do have some more general questions that would be nice to address:

Based on these results, can the authors make any predictions regarding the minimum size/weight that might be able to achieve sustained flight? As scale continues to decrease, and Re goes down, drag continues to rise - what is the theoretical limit.... is there a limit of the required (relative) muscle mass? (although the larger species also have very high fractions of muscle mass - Fig 7B)

Reply 4.2. We thank the reviewer for raising this broader question. Our data show that viscosity-induced increases in drag impose progressively stronger constraints on further miniaturization in Diptera, but they do not allow us to predict a strict minimum viable body size for sustained flight. In the revised manuscript, we clarify that insects below ~2 mg operate in a regime where decreasing Reynolds number increases relative drag and limits further gains in aerodynamic economy, even though absolute aerodynamic power remains small (Discussion, lines 883-890). Here, we also added a short section noting that, beyond Diptera, viscosity may limit flight performance in the smallest specialized fliers that operate deep in the laminar-viscous Reynolds-number regime.

We therefore interpret our results as consistent with progressive physical limits on further size reduction rather than evidence for a hard lower bound on the size for powered flight. We hope the revised Discussion reflects this more clearly.

Comment 4.3. The outlier continually demonstrated by Culicomorpha has always fascinated me - it is the cousin that no one can believe they are related to! - Is there anything more that can be said about the pressures that might have led to this evolutionary line? There is a passing comment in the final lines of the paper, but is echoed in the abstract, that reproductive success is here more important than aerodynamic efficiency - what does this refer to?

Reply 4.3. We fully agree with the reviewer that the striking divergence of Culicomorpha compared to other Diptera is one of the most intriguing aspects of our study. As requested, we therefore expanded the corresponding section of the Discussion to clarify the evolutionary pressures that may explain this lineage's distinct biomechanical phenotype (lines 927-953). Our data show that Culicomorpha combine extremely high wingbeat frequencies with low stroke amplitudes and elevated aerodynamic and acoustic power. These traits are consistent with selection acting on acoustic signalling during swarm-based mate localization, where reproductive success depends on producing and detecting flight tones. This emphasis on acoustic performance likely contributes to their departure from the kinematic and aerodynamic patterns observed in other Diptera.

Academic editor comment:

Comment E.1 I do agree that the paper is worth publishing. This is because such studies combining a range of fields, from phylogeny and functional morphology to biomechanics and physics are extremely hard to execute and therefore quite rare. They also are important for gaining a more comprehensive picture and bridging fields. The decision to revise substantially in four months is reasonable. The authors should still try as hard as they can to address all comments, including R1's, or rebut if necessary.

Reply E.1 We appreciate the Editor's positive evaluation of the manuscript and the acknowledgment of the breadth and difficulty of this type of integrative study. We have carefully addressed all reviewer comments, including the detailed points raised by Reviewer 1, and have revised the manuscript accordingly. Where necessary, we added improved clarifications, new analyses and corresponding figure, and a dedicated Scope & Limitations section to ensure that our claims remain well supported and appropriately framed. We trust that these revisions fully satisfy the concerns raised during review and strengthen the manuscript substantially.

Comment E.2 I have one request of my own and hope the authors have the data:

When invoking sexual selection to explain a pattern, it is necessary to collect sex-specific data. It would be very useful to see if males and females of mosquitos and crane flies show different patterns of wing beat and acoustic production. The expectation here is that males are the ones chasing females and therefore the one looking for signs that guide them to their location. Often these traits are sexually dimorphic and could manifest through differences in the patterns shown by the sexes. My question to the authors is whether they can trace the sexes back in their data and look for differences to support the claims of sexual selection being involves.

Reply E.2 We thank the Academic Editor for this very relevant point. In our comparative dataset, the sampling strategy maximised phylogenetic and size breadth rather than sex-balanced sampling. However, for taxa where sexual dimorphism is strong and directly relevant to acoustic mate localization (most notably Culicomorpha) we systematically used males, because males form swarms and are the phonotactically active sex that detect and pursue females via their wingbeat tones (Gibson and Russell, 2006; Gupta et al., 2024; Pennetier et al., 2010). This is now explicitly stated in the revised Methods (lines 178-184).

Male–female acoustic interactions in Culicomorpha are intrinsically coupled: males adjust their wingbeat to track the interference (difference) tone arising between their own wingbeat and the female's. Both sexes show characteristic adaptations in wingbeat kinematics and auditory tuning associated with this behaviour, as documented in the literature (e.g. Gibson & Russell 2006; Pennetier et al. 2010; Gupta et al. 2024). Because these dynamics are fundamentally interactive, single-individual recordings cannot capture the full, sex-specific acoustic loop that governs close-range mating behaviour. Rigorous testing of sexual-selection hypotheses therefore requires paired (male–female) co-flight experiments with concurrent kinematic and acoustic tracking; an approach that lies beyond the scope of this broad comparative study.

Importantly, the presence of sex-linked wingbeat–acoustic adaptations in both sexes means that the male aerodynamic and acoustic phenotype remains informative for assessing the evolutionary trade-off between enhanced signalling and flight cost, which is precisely the comparative question addressed here. We now state this more clearly in the revised Discussion section (lines 927-953).

Following the Editor's recommendation and Reviewer 1's suggestions, we have also tempered causal language, describing sexual selection as a plausible contributor to the Culicomorpha divergence rather than an exclusive driver (lines 927-953). Finally, the newly added Scope & Limitations paragraph clarifies that fully resolving sex-specific and interaction-dependent aero-acoustic processes requires the targeted experimental work we are currently developing with auditory specialists (lines 955-965).

We hope that these clarifications address the Editor's concern and keep the manuscript's conclusions well supported by the available data.

Comment E.3 Remark about image copyright.

Reply E.3 Regarding the inquiry concerning image copyright and ownership: to ensure full compliance and data transparency, we have replaced the insect images in our Figures with original frames extracted from our own high-speed video recordings. These frames specifically illustrate the specimens during flight, providing an authentic representation of the kinematics discussed.

References

Gibson, G. and Russell, I. (2006). Flying in Tune: Sexual Recognition in Mosquitoes. *Current Biology* **16**, 1311–1316.

Gupta, S., Cribellier, A., Poda, S. B., Roux, O., Muijres, F. T. and Riffell, J. A. (2024). Mosquitoes integrate visual and acoustic cues to mediate conspecific interactions in swarms. *Current Biology*.

Hoerner, S. F. (1965). Fluid-dynamic drag: practical information on aerodynamic drag and hydrodynamic resistance.

Le Roy, C., Tervelde, N., Engels, T. and Muijres, F. T. (2025). Adaptations in wing morphology rather than wingbeat kinematics enable flight in small hoverfly species. *Elife* **13**,.

Lyu, Y. Z., Zhu, H. J. and Sun, M. (2019). Aerodynamic forces and vortical structures of a flapping wing at very low Reynolds numbers. *Physics of Fluids* **31**,.

Pennetier, C., Warren, B., Dabiré, K. R., Russell, I. J. and Gibson, G. (2010). "Singing on the wing" as a mechanism for species recognition in the malarial mosquito Anopheles gambiae. *Curr. Biol.* **20**, 131–6.
