# Supplementary material for: Dipteran flight diversity is shaped by aerodynamic constraints, scaling, and evolutionary trade-offs
Source: PLoS Biol. 2026 Jul 9;24(7):e3003473. doi: 10.1371/journal.pbio.3003473 (PMC13349162; doi:10.1371/journal.pbio.3003473)
Supplement: Attachment — Submitted filename: responses_to_reviewers_round_2.pdf [file pbio.3003473.s011.pdf]

Reviewer #1:

Comment 1.1. This remains an impressive body of work. Any quibbles I have with language, framing or detail in the main body are unimportant as the description of process is thorough, and readers will have the opportunity to reanalyse/ reframe in their own interest. I strongly appreciate the efforts to make the data usefully available.

Reply 1.1. We thank the reviewer for their detailed reviews, positive evaluation of our study, and for helping us further improve our study. We addressed all final comments as described below, and hope that the revised manuscript is now fit for publications.

Comment 1.2. One main issue remains of concern – largely concerning the framing/hypotheses.

Constraint cannot be inferred.

Abstract: "Combining these data with our scaling analyses suggests that tiny Diptera are primarily constrained by aerodynamic force production, and maintain weight support through relatively larger wings and increased wingbeat frequencies. In contrast, as Diptera increase in size, hovering flight becomes progressively constrained by power availability, resulting in markedly elevated relative flight-muscle mass among the largest species."

Disagree. No constraint can be inferred. Aerodynamic force requires power and area. Sufficient power and area is observed – the flies manage to hover. If you took a little bit of the area away from either case (and left all else unchanged), weight would not be supported: force is limiting. If you took a little bit of the power away (and left all else unchanged), weight would not be supported: power is limiting. In both cases. If you found that one of them had a high power margin (so could power an acceleration upwards) they would also have a high lift margin (so could provide the force needed to accelerate upwards).

That small flies have 'relatively' large wings, and big flies have 'relatively' big muscles is not untrue or uninteresting, but may simply reflect issues with how 'relatively' is defined.

Reply 1.2. We thank the reviewer for this careful clarification and fully agree that these constraints cannot be inferred directly from our data. As the reviewer correctly notes, aerodynamic force production and mechanical power availability are jointly required for hovering flight in all species, and neither can be identified as uniquely limiting based on successful weight support alone.

In the original abstract, our wording overstated this point by implying distinct limiting constraints at opposite ends of the size range. We have therefore revised the abstract to remove any inference of constraint, and to reframe our results in terms of size-dependent trait combinations that meet shared physical requirements for hovering flight. The revised abstract now states that maintaining weight support across the Dipteran size range requires systematic allometric adjustments in wing morphology, wingbeat kinematics, and flight musculature. Hereby, smaller and larger Diptera achieves this through different combinations of these traits. Specifically, smaller Diptera employ relatively larger wings and higher wingbeat frequencies, whereas larger Diptera exhibit increased relative flight musculature to sustain the required mechanical power output.

In addition, we revised relevant text in the Introduction, Results, and Discussion sections to also here avoid language that could imply size-dependent force or power limitations. We hope this revised framing addresses the reviewer's concern and more accurately reflects what can be concluded from the data.

Comment 1.3. Minor points.

I get a bit confused about assumptions relating to geometric, kinematic and dynamic similarity. I know what geometric similarity is: lengths scale with L, areas with L^2, volumes with L^3, second moments of area (relevant for lift) L^4 and third moments of area (relevant for power) L^5. Kinematic similarity in the context here presumably relates to consistent angles but does not have a time element to it (this could be clarified – kinematics sometimes covers velocities, accelerations too). The dynamic similarity being discussed in this paper relates only to Reynolds number (dynamic similarity of fluids); it may be worth highlighting that this is not the same as would be expected from wave- or terrestrial motions (e.g. Froude number), which relates to the time scaling required to achieve kinematic similarity (e.g. due to ballistic paths in running gaits). If that definition was applied, dynamic similarity would be achieved whenever the centre of mass was supported in steady hover.

Reply 1.3. We thank the reviewer for highlighting the need for clearer definitions of geometric, kinematic, and dynamic similarity. The reviewer is correct that we define these as:

For **Geometric similarity**, all linear dimensions scale with a characteristic length L, such that areas scale with L^2, volumes with L^3, and higher-order moments accordingly.

Kinematic similarity is defined that the geometry of motion is preserved across sizes, specifically that stroke amplitude, angles of attack, and phase relationships remain constant. Under this definition, time is not rescaled, and wingbeat frequency therefore remains constant when expressed in absolute units. We agree that kinematics can in some contexts include

velocities and accelerations, and we now clarify in the manuscript that here the term refers strictly to similarity of wingbeat angles and motion patterns, not time scaling.

By **Dynamic similarity**, we refer exclusively to fluid dynamic similarity, such that the relevant dimensionless airflow parameters are constant across sizes. Because our analyses focus on hovering flight, forward speed is negligible and thus Strouhal number is not informative. As a result, Reynolds number is the primary dimensionless parameter governing dynamic similarity in our analysis.

We agree with the reviewer that this definition differs from those commonly used in terrestrial or wave-dominated locomotion, where Froude number governs similarity through the balance of inertial and gravitational forces. In our framework, assuming dynamic similarity (i.e. Reynolds-independent aerodynamics) represents a hypothetical baseline rather than a biological expectation, and is used solely as a reference against which to evaluate the observed Reynolds-dependent scaling of aerodynamic forces and power in real insects.

To address this explicitly, we added dedicated paragraphs defining geometric, kinematic, and dynamic similarity to both the revised Introduction and Method sections (lines 142-147 and 382-397, respectively).

Comment 1.4. Check throughout: should Dipteran be dipteran (unless beginning sentence) – in my training it would have been 'dipterans are Diptera.

Reply 1.4. We thank the reviewer for pointing this out. We have revised the manuscript to use terminology consistently, following standard zoological convention: Diptera is used for the taxonomic order, treated as a plural noun, whereas dipteran (singular or plural) is used as a common noun or adjective when referring to individual species or traits (e.g. "dipteran flight", "dipterans"). Capitalization has been corrected accordingly throughout the manuscript.

Comment 1.5. "Insects are the most species-rich group of animals"
Untrue (in detail): arthropods are surely an even more species-rich group of animals.

Reply 1.5. We have revised the sentence to state that insects are the most species-rich *class* of animals, thereby addressing the distinction from arthropods as the most species-rich phylum.

Comment 1.6. L51 (Back to the kinematics query)
"One important effect is that, under geometric similarity, the ability of flapping wings to generate sufficient aerodynamic force for weight support decreases with decreasing body size. Tiny insects

need to compensate for this through allometric adjustments in wing…"

Disagree. Under geometric similarity mg= constant.L^4 f^2 (see ref [20] etc.). So tiny insects 'could' generate sufficient force with just increase in frequency. They do have higher frequencies, but not enough: I agree they do have relatively (relative to mass^1/3) larger wings, and that is interesting – just reframe.

Reply 1.6. We agree with the reviewer that, under geometric similarity, sufficient aerodynamic force for weight support could in principle be achieved by increasing wingbeat frequency alone, and that our original wording was thus incorrect.

We therefore have revised the text accordingly. The manuscript now states that, under geometric similarity, aerodynamic force production does not scale in proportion to body weight across sizes, and that maintaining weight support at small body sizes requires allometric adjustments in wing morphology and/or wingbeat kinematics. This revised framing avoids implying an inherent force limitation and is consistent with the reviewer's point (see also Reply 1.2).

Comment 1.7. L. 62 "In the inertial regime, drag is relatively low and approximately independent of Reynolds number (D ∝ Re0)"

Untrue. Drag (absolute) scales with the square of length and speed: Re^2. And do you have a citation for that? Drag coefficient certainly scales less strongly than at low Re, but I recall Re^-0.2 being mentioned, probably in Hoerner.

Line 67. Again, drag coefficient is intended.

L. 68. Not drag absolute – coefficient intended. Also probably true for body and waiting to be determined for wings – depends on angle of incidence, and that is a story that comes later.

Reply 1.7. We thank the reviewer for pointing out this incorrect wording. The reviewer is correct that absolute drag scales with the square of characteristic length and velocity, and therefore with Reynolds number squared. Our intention in this section was to refer to the drag coefficient, which approaches Reynolds-number independence in the high-Re inertial asymptotic regime.

We have revised the paragraph accordingly to explicitly refer to the drag coefficient rather than absolute drag throughout, and to clarify that approximate Reynolds-number independence applies only in this inertial asymptotic limit (lines 56-70).

Comment 1.8. Check you with to use superscript * to indicate both weight specific and lift coefficient specific.

Reply 1.8. As requested, we have ensured consistent use of the superscript "$*$" throughout the manuscript to indicate normalized quantities. Specifically, superscript "$*$" is now used systematically for both weight-normalized quantities (e.g. $P^* = P/mg$) and lift-normalized aerodynamic coefficients (e.g. $D^* = C_D/C_L = D/L$). Because in steady hovering flight lift equals weight ($L = mg$), these two normalizations are equivalent in the present context. This notation is now applied consistently across the text, equations, and figure captions.

Reviewer #3:

Comment 3.1. The authors have done a very thorough job of addressing my concerns with their initial submission. I am happy to recommend publication.

Reply 3.1. We thank the reviewer for their time and effort put into reviewing our paper, and their positive assessment of our revised manuscript.

Editor:

Comment E.1. This remains an impressive body of work. Any quibbles I have with language, framing or detail in the main body are unimportant as the description of process is thorough, and readers will have the opportunity to reanalyse/ reframe in their own interest. I strongly appreciate the efforts to make the data usefully available.

Thank you for your patience while we considered your revised manuscript "Diptera flight diversity is shaped by aerodynamic constraints, scaling, and evolutionary trade-offs" for publication as a Research Article at PLOS Biology. This revised version of your manuscript has been evaluated by the PLOS Biology editors, the Academic Editor, and two of the original reviewers.

Based on the reviews and on our Academic Editor's assessment of your revision, we are likely to accept this manuscript for publication, provided you satisfactorily address the remaining points raised by the reviewers, and the following data and other policy-related requests.

Reply E.1. We thank the Editor for the positive evaluation of our manuscript and for the encouraging assessment of the rigor of our approach and the transparency of our methods and data sharing. We greatly appreciate the Editor's supportive comments and the opportunity to revise the manuscript accordingly, and we are pleased to have addressed the remaining reviewer and policy-related requests. We hope that the revised version now fully meets the standards for publication as a Research Article in *PLOS Biology*.

Comment E.2. a) Regarding the Title, we wonder whether it would be better to put "Dipteran" as an adjective describing "flight diversity" rather than "Diptera" as the noun for the order of insect?

Reply E.2. We agree with the Editor that using "Dipteran" as an adjective provides clearer and more conventional phrasing. Thus, we have revised the title accordingly.

Comment E.3. b) Please attend to the remaining requests from reviewer #1.

Reply E.3. We have attended to all remaining requests from Reviewer #1. In particular, we revised the manuscript to clarify the framing of similarity and scaling assumptions, corrected references to drag versus drag coefficients, ensured consistent use of notation, and adjusted terminology and phrasing throughout for accuracy and consistency. We believe these revisions fully address the reviewer's comments.

Comment E.4. c) Please address my Data Policy requests below; specifically, we need you to supply the numerical values underlying Figs 1ABC, 2ABCDEF, 3CDEFGHIJ, 4ABCDEFGH, 5ABCDE, 6ABC, 7ABC, 8AB, S1AB, S2, S3AB, either as a supplementary data file or as a permanent DOI'd deposition. I note that you already have an associated DRYAD deposition (DOI: 10.5061/dryad.gxd25480s), but this currently only contains raw data. Please could you complete this deposition with the data and code underlying the Figures?

Reply E.4. The numerical data underlying all main and supplementary figures were already deposited in the associated Dryad repository (DOI: 10.5061/dryad.gxd25480s). We have now updated this repository to additionally include all custom code used to generate the manuscript figures.

We have also added a statement to all figure legends indicating that the data underlying each figure are available in an online repository, with full details provided in the Data Availability Statement.

Comment E.5. d) Please cite the location of the data clearly in all relevant main and supplementary Figure legends, e.g. "The data underlying this Figure can be found in S1 Data" or "The data underlying this Figure can be found in DOI: 10.5061/dryad.gxd25480s"; also update your Data Availability Statement accordingly.

Reply E.5. We have revised all relevant main and supplementary figure legends to explicitly state that the data underlying each figure are available in our online repository, with full details provided in the Data Availability Statement. In addition, we have updated the Data Availability Statement to clearly describe the location of the numerical data and associated custom code in the Dryad Digital Repository (DOI: 10.5061/dryad.gxd25480s).

Comment E.6. e) Please make any custom code available, either as a supplementary file or as part of your data deposition.

Reply E.6. As also mentioned in Reply E.4, we now made all custom code required to generate the figures publicly available, by adding these to the existing Dryad data deposition (DOI: 10.5061/dryad.gxd25480s). The version of our in-house CFD code WABBIT that has been used to carry out the numerical simulations is archived on ZENODO with the DOI https://doi.org/10.5281/zenodo.20234346. This is now mentioned explicitly in the Data Availability Statement.

Comment E.7. f) Please include the URLs of your funders in the Financial Disclosure statement.

Reply E.7. As requested, we added URLs of the funding bodies to the revised Funding section and Financial Disclosure statement.

Comment E.8. As you address these items, please take this last chance to review your reference list to ensure that it is complete and correct. If you have cited papers that have been retracted, please include the rationale for doing so in the manuscript text, or remove these references and replace them with relevant current references. Any changes to the reference list should be mentioned in the cover letter that accompanies your revised manuscript.

Reply E.8. We have carefully reviewed the full reference list to ensure that it is complete, accurate, and up to date.
