# Supplementary material for: Dipteran flight diversity is shaped by aerodynamic constraints, scaling, and evolutionary trade-offs
Source: PLoS Biol. 2026 Jul 9;24(7):e3003473. doi: 10.1371/journal.pbio.3003473 (PMC13349162; doi:10.1371/journal.pbio.3003473)
Supplement: Peer review history

# Editor report: Decision Letter 0

**DOI:** 10.1371/journal.pbio.3003473.r001

**Authors:** Roland Roberts

17 Oct 2025

Dear Dr Muijres,

Thank you for submitting your manuscript entitled "Diptera flight diversity is shaped by aerodynamic constraints, scaling, and evolutionary trade-offs" for consideration as a Research Article by PLOS Biology.

Your manuscript has now been evaluated by the PLOS Biology editorial staff as well as by an academic editor with relevant expertise, and I'm writing to let you know that we would like to send your submission out for external peer review. Please accept my apologies for the delay incurred while we obtained external expert advice.

However, before we can send your manuscript to reviewers, we need you to complete your submission by providing the metadata that is required for full assessment. To this end, please login to Editorial Manager where you will find the paper in the 'Submissions Needing Revisions' folder on your homepage. Please click 'Revise Submission' from the Action Links and complete all additional questions in the submission questionnaire.

Once your full submission is complete, your paper will undergo a series of checks in preparation for peer review. After your manuscript has passed the checks it will be sent out for review. To provide the metadata for your submission, please Login to Editorial Manager (https://www.editorialmanager.com/pbiology) within two working days, i.e. by Oct 21 2025 11:59PM.

If your manuscript has been previously peer-reviewed at another journal, PLOS Biology is willing to work with those reviews in order to avoid re-starting the process. Submission of the previous reviews is entirely optional and our ability to use them effectively will depend on the willingness of the previous journal to confirm the content of the reports and share the reviewer identities. Please note that we reserve the right to invite additional reviewers if we consider that additional/independent reviewers are needed, although we aim to avoid this as far as possible. In our experience, working with previous reviews does save time.

If you would like us to consider previous reviewer reports, please edit your cover letter to let us know and include the name of the journal where the work was previously considered and the manuscript ID it was given. In addition, please upload a response to the reviews as a 'Prior Peer Review' file type, which should include the reports in full and a point-by-point reply detailing how you have or plan to address the reviewers' concerns.

During the process of completing your manuscript submission, you will be invited to opt-in to posting your pre-review manuscript as a bioRxiv preprint. Visit http://journals.plos.org/plosbiology/s/preprints for full details. If you consent to posting your current manuscript as a preprint, please upload a single Preprint PDF.

Feel free to email us at plosbiology@plos.org if you have any queries relating to your submission.

Kind regards,

Roli Roberts

Roland Roberts, PhD

Senior Editor

PLOS Biology

rroberts@plos.org

# Peer review: Decision Letter 1

**DOI:** 10.1371/journal.pbio.3003473.r002

**Authors:** Roland Roberts

17 Dec 2025

Dear Dr Muijres,

Thank you for your patience while your manuscript "Diptera flight diversity is shaped by aerodynamic constraints, scaling, and evolutionary trade-offs" was peer-reviewed at PLOS Biology. It has now been evaluated by the PLOS Biology editors, an Academic Editor with relevant expertise, and by four independent reviewers.

You'll see that reviewer #1 says that the dataset is outstanding and of broad interest. However, s/he thinks that the presentation needs substantial improvement, questions the novelty and reliability of some claims, and suggests that you have omitted some obvious mechanisms. S/he presents a substantial list of issues to attend to. Reviewer #2 is very positive, but stresses that s/he is not a flight expert. S/he also has problems with the presentation, and has a number of points for discussion. Reviewer #3 is also impressed, but wonders if the paper could be re-focussed and re-structured to make it an easier read, and has a list of requests for clarification. Reviewer #4 regrets that s/he did not have enough time, but likes the study and has two points for discussion. Please note that the Academic Editor has also included a request (see the foot of the email).

In light of the reviews, which you will find at the end of this email, we would like to invite you to revise the work to thoroughly address the reviewers' reports.

Given the extent of revision needed, we cannot make a decision about publication until we have seen the revised manuscript and your response to the reviewers' comments. Your revised manuscript is likely to be sent for further evaluation by all or a subset of the reviewers.

In addition to these revisions, you will need to complete some formatting changes, which you will receive in a follow up email. A member of our team will be in touch with a set of requests shortly.

We expect to receive your revised manuscript within 4 months. Please email us (plosbiology@plos.org) if you have any questions or concerns, or would like to request an extension.

At this stage, your manuscript remains formally under active consideration at our journal; please notify us by email if you do not intend to submit a revision so that we may withdraw it.

**IMPORTANT - SUBMITTING YOUR REVISION**

Your revisions should address the specific points made by each reviewer. Please submit the following files along with your revised manuscript:

1. A 'Response to Reviewers' file - this should detail your responses to the editorial requests, present a point-by-point response to all of the reviewers' comments, and indicate the changes made to the manuscript.

*NOTE: In your point-by-point response to the reviewers, please provide the full context of each review. Do not selectively quote paragraphs or sentences to reply to. The entire set of reviewer comments should be present in full and each specific point should be responded to individually, point by point.

You should also cite any additional relevant literature that has been published since the original submission and mention any additional citations in your response.

2. In addition to a clean copy of the manuscript, please also upload a 'track-changes' version of your manuscript that specifies the edits made. This should be uploaded as a "Revised Article with Changes Highlighted" file type.

*Re-submission Checklist*

When you are ready to resubmit your revised manuscript, please refer to this re-submission checklist: https://plos.io/Biology_Checklist

To submit a revised version of your manuscript, please go to https://www.editorialmanager.com/pbiology/ and log in as an Author. Click the link labelled 'Submissions Needing Revision' where you will find your submission record.

Please make sure to read the following important policies and guidelines while preparing your revision:

*Published Peer Review*

Please note while forming your response, if your article is accepted, you may have the opportunity to make the peer review history publicly available. The record will include editor decision letters (with reviews) and your responses to reviewer comments. If eligible, we will contact you to opt in or out. Please see here for more details:

https://blogs.plos.org/plos/2019/05/plos-journals-now-open-for-published-peer-review/

*PLOS Data Policy*

Please note that as a condition of publication PLOS' data policy (http://journals.plos.org/plosbiology/s/data-availability) requires that you make available all data used to draw the conclusions arrived at in your manuscript. If you have not already done so, you must include any data used in your manuscript either in appropriate repositories, within the body of the manuscript, or as supporting information (N.B. this includes any numerical values that were used to generate graphs, histograms etc.). For an example see here: http://www.plosbiology.org/article/info%3Adoi%2F10.1371%2Fjournal.pbio.1001908#s5

*Blot and Gel Data Policy*

We require the original, uncropped and minimally adjusted images supporting all blot and gel results reported in an article's figures or Supporting Information files. We will require these files before a manuscript can be accepted so please prepare them now, if you have not already uploaded them. Please carefully read our guidelines for how to prepare and upload this data: https://journals.plos.org/plosbiology/s/figures#loc-blot-and-gel-reporting-requirements

*Protocols deposition*

To enhance the reproducibility of your results, we recommend that if applicable you deposit your laboratory protocols in protocols.io, where a protocol can be assigned its own identifier (DOI) such that it can be cited independently in the future. Additionally, PLOS ONE offers an option for publishing peer-reviewed Lab Protocol articles, which describe protocols hosted on protocols.io. Read more information on sharing protocols at https://plos.org/protocols?utm_medium=editorial-email&utm_source=authorletters&utm_campaign=protocols

Thank you again for your submission to our journal. We hope that our editorial process has been constructive thus far, and we welcome your feedback at any time. Please don't hesitate to contact us if you have any questions or comments.

Sincerely,

Roli Roberts

Roland Roberts, PhD

Senior Editor

PLOS Biology

rroberts@plos.org

------------------------------------

REVIEWERS' COMMENTS:

Reviewer #1:

This manuscript summarises a considerable amount of data. In this context it is certainly outstanding in its field, and will be of interest quite broadly, covering evolution, entomology, aerodynamics and acoustics.

I have three main issues:

1.The loss of exciting/ informative / important detail with the presentation (consider Figs 3 and 5D,E - lots of indistinguishable wiggly lines). I cannot currently assess the data availability. It would be nice to be able to compare and contrast any pair of measured insects from the PCs of the wing shapes and the Fourier terms for the kinematics. It should be pretty simple for me to recreate the SI movie (which is excellent) using measured - and reported - summarising statistics. Without this, (and I wouldn't want to go right back to early analysis) I am reliant on the story-telling as told, which I think misses much of the potential value of this study.

2.The novelty and security of the take-home messages. Was it already known that craneflies were quite big and relatively slow-flapping? (Ellington certainly did some craneflies?). That robberflies had big muscles? That mosquitoes flapped with a high frequency, low amplitude, high incidence that didn't make sense in terms of aerodynamic power… but might (might) relate to sexual selection? That flappers with high aerodynamic power for their size (see 'efficiency' note below) also have high acoustic power for their size?

3.The omission of some obvious mechanisms and sidestepping of analysis / logical flow.

Efficiency is not efficiency, or even 'aerodynamic efficiency' (per Rankine Froude). In the context of this paper this is a semantic/technical point. Useful power / Total power… in hovering, there is no useful power, so there is always zero efficiency. One aerodynamic efficiency applied to hovering considers the minimum power that would have been possible (for rotorcraft, this relates to disc area, weight, density etc.) as being 'useful'. The appropriate swept area is not obvious for flapping, and isn't really of interest. So I am happy for W/N to be used for the metric for economy (but I am not surprised that this would scale with size). A species with a high aerodynamic power demand for hovering may reasonably be viewed as being less 'economical' (I avoid the term 'efficient' for history above, but will cope if necessary) than a species of the same mass that has a lower aerodynamic power (for hovering). There is a spread of these economies (Fig. 7a). But note that - without the distraction of the lines - the spread is approximately even across scales, and there is a negligible relationship with mass (especially noting semi-log scale). So the focus here is on the relatively high power of the mosquitoes - reasonably related to their small amplitudes, high angles; but the same question arises at the larger end. Presumably the high-power demand large insects have relatively short wings - and this relates to induced power (see below).

The scaling discussion may end up invoking more interacting terms than can be handled simply here. Most notable is the omission of induced power. More later.

The propeller approach taken here is an adaptation of the quasi-steady blade-element analysis (see Ellington etc.)… but it entirely omits the induced power/drag aspect. To some extent this is fine as induced effects can be wrapped up into the D* metric (Fig 5) - but it does need to be mentioned if the high D* of the normal looking flies (sorry, cartoon looks a bit like a blowfly?) is going to be accounted for in comparison with the craneflies.

Is viscosity the answer (Fig 5F)? And is it actually needed as an answer? It is not clear that there is an up-tick in aerodynamic power at small masses. The effect of viscosity would be fairly simple to determine by playing small and large insects through the CFD at high and low Re ('wrong') respectively - does the power shift as expected from the simplified viscosity account?

Note that D* around 1 is expected for a thin flat plate at 45degrees to the flow: without leading-edge suction, force will be approximately perpendicular to the surface. Alpha is not reported… does it relate to CD:CV throughout the flap?

The power calculations are couched in propeller terminology, with P* approx. Ud.D*. This approach entirely misses the possibility of reduced power demand through wake recapture: as can be seen from Fig 5D there are relatively high forces at the limits of downstroke/upstroke in the high frequency flappers, when the wing is moving slowest. This energetic benefit to wake recapture (see, for instance, some of Fritz Lehmann's work) would not be accounted for with the propeller analysis. I do not expect that mosquitoes will suddenly become low-power hoverers, but it is an obvious flaw with the approach. I expect the 'validation' referred to (L.397) might indicate a greater discrepancy between CFD and propeller reduction for the mosquitoes (CFD expected to calculate lower power due to the mechanisms above). A touch more on the validation would be comforting: even if it is only 'typical mosquito, typical Drosophila, typical cranefly, typical blowfly' to cover economical/uneconomical at both ends of the mass scale. Systematic error is not disastrous to the analysis, but it would be nice if it was small enough to be neglected.

How fair is it to infer that hovering with a relatively high aerodynamic power indicates low mechanical efficiency generally? 1) Other mechanical power demands may be considerable. Inertial power is worth a mention, even if it is then assumed that it can be neglected for some reason. It is one cost sometimes used to account for the general inefficiency of hovering in all sorts of animals. 2) Hovering does not represent all flight; it may be that an albatross hovers appallingly. Specialisation for sprinting, load-carrying, manoeuvring… would all demand compromise with hovering economy. While the sexual selection hypothesis for the peculiar mosquitoes is not unreasonable, others (particularly load carrying) are not precluded. While the measurements here are nice, they have not particularly shifted the debate.

Specifics.

I cannot agree that the data indicates a limit to small size: an unlabelled Figure 7 does not show higher aerodynamic power at the smallest size compared with the largest. A 'plateau in reduction' is pretty subjective. See above for ways of testing influence of viscosity.

I cannot agree that 'Scaling analysis show that smaller species compensate for reduced aerodynamic force production efficacy…'. A scaling analysis of hovering that does not include induced power is likely to be misleading. From memory, a Rankine-Froude derivation for induced power in hover would indicate an increased mass specific power demand with size (with isometric scaling of wing length) - as m^1/6. See Ellington 1984 (later papers).

But as above, I think the scaling analysis might best be avoided. There is enough data to compare different species of same mass.

To conclude: lovely data, I trust it will become available in a useful form; scaling analysis problematic due to induced power (probably means intractable here); power calculations miss wake-recapture, but effect likely small (though would be nice to be quantified) and inertial power (to be acknowledged then dodged); uneconomical big flies need an account (I would point to induced power); viscosity argument to be validated; sexual selection argument for mosquitoes not significantly advanced (though interesting and worth considering).

Reviewer #2:

This paper examines the biomechanics and biophysics of flight in flies, integrating a variety of approaches. The paper is a methodological tour de force, ranging from phylogenetic comparative analysis, to flight kinematics, to geometric morphometrics, and acoustics. Moreover, nearly each analysis is rooted in first principles theory. I really appreciated the comparative approaches taken here, while most biomechanical studies focus on a single (or small number) of species. Here, we get a view across a megadiverse clade at what is similar and what is different across all this diversity, with insights to explain these patterns. I commend the authors on this detailed work, although I will admit I am not an expert on flight biophysics so hopefully other reviewers can judge the highly technical bits. I have some suggestions to improve the study.

Comments:

There are a lot of analyses and results presented, and this comprehensiveness is a strength of the paper, but also can make it a bit hard to extract the key big picture conclusions. I don't have specific recommendations, but it would be nice to sharpen the key take-away points of general interest.

I found myself wondering how what we found is different than what we could have found. For example, could we have found that shape is highly conserved, but flies diversify their kinematics to have different flight abilities? Highlighting these also helps drive home the key points.

The differences between the mosquitos, craneflies, and the rest of flies is very intriguing. The authors attribute this to the need to use acoustic signaling for sexual selection (at least in part). Is it possible there was some key morphological-physiological innovation not visible in external wing shape that could have both allowed higher performance and at the same time constrained diversification within the larger radiation excluding these other lineages?

I was missing some broader context about how this study fits into what we know about insect and other animal flight. Are there lessons here that may generate hypotheses for other groups? For example, if we did this same study for Hymenoptera could we pose some hypotheses based on flies? Some forward looking comments will be welcome. I am not a flight or fly expert, but all the more so I am curious how this compares with the scaling other groups including mammal and bird flight and what universalities might exist.

L15 Basal- Calling species that are part of a more species-poor branch diverging early from the rest of the clade "basal" is a bit frowned upon now because it can be misleading (along the lines of the fallacy that some species alive today are ancestors of other species). Do we have evidence that the ancestors at the base of the tree were more mosquito-like? This would require some knowledge of the fossil record (which I don't have for flies).

L48: "evolutionary pressure forces". Maybe you mean, "evolutionary pressures"?

L68: resultS

L413 missing space

L492: modeS

L745 (paragraph): could this be interpreted as "many-to-one mapping" as is found in fish jaws, for example? i.e. there are many different morphologies that produce the same function. If so, it could help connect to other literatures.

759: conflates "early branching" lineages with "early Diptera". It is not shown that the ancestral phenotype looked like mosquitoes or craneflies. Indeed Fig 2 seems to suggest their wings did not.

L800: How can morphology be not constrained within younger clades but constrained on larger phylogenetic scales, since the former are nested within the latter? Clarify.

Reviewer #3:

In this comprehensive paper, Le Roy et al. explore the relationship between wing morphology, flight ability, and body size across a diversity of dipterans. First, they captured body size and wing morphology (size and shape) for 133 species. They then used a high-speed camera to determine the flight kinematics (e.g., wingbeat frequency, angle of attack, amplitude) of a subset of 46 species. Next, they used the morphological and kinematic data to model flight using Computational Fluid Dynamics, generating aerodynamic parameters such as lift and drag forces and aerodynamic power. Using these parameters, they explored how wing morphology, kinematics, and aerodynamics scale with body size and co-vary across phylogeny. Finally, they integrated the data to explore compensation mechanisms in small species and a possible trade-off between flight and acoustic power in mosquitoes. The amount of data collected is hugely impressive, and the data processing and analysis are well described (albeit with some caveats described below). I really enjoyed this paper and think it will be of interest to anyone interested in the evolution of insect wings, flight biomechanics, and the scaling of functionally well-elucidated morphology.

That being said, I have a number of comments that the authors should address.—

1.This is a very long paper (~20,000 words), and very detailed, which makes it more challenging to follow than necessary. The introduction could be trimmed down substantially, and most of the methods could be moved to the supplementary material. I do not work in flight biomechanics, and while I could follow the methodology, at times it was difficult to see the wood for the trees—that is, the details obscured the hypotheses being tested. It would be clearer to focus on which parameters are assessed at each stage of analysis (geometric parameters from the morphological analysis, kinematic parameters from the high-speed camera, and dynamic parameters from CFD). The focus should then be on the biological meaning of these parameters and how they relate to the hypotheses, rather than on the method by which they were collected. This structure (morphological, kinematic, and dynamic) is laid out clearly in Figure 4, but not consistently elsewhere in the paper.

2.More generally, the paper should be more firmly structured around specific hypotheses (e.g., the relationship between wings used for flight versus wings used for sound production; the impact of small body size on wing shape and function). The introduction explicitly positions the dataset as essential for understanding the selective pressures shaping insect flight and flight morphology, yet these selective pressures are not very clearly articulated. As written, the reader is somewhat overwhelmed—though also impressed—by the sheer quantity of data.

3.Figure 4. The PGLS was fit to log-transformed data (L480), yet the y-axes are sometimes log-transformed (e.g., Fig. 4A) and sometimes not (e.g., Fig. 4C). Further, based on Table 1, the PGLS was a single-predictor linear regression with a slope and intercept. How, then, does the PGLS in Figure 4D appear curved when plotted on a log-log scale? Similarly, if there is a linear relationship between the logs of x and y, why are the fits not curved when plotted on a log-linear scale (e.g., Fig. 4G)?

4.Equation (11) relies on an assumption that is not fully addressed. The assumption is that as the observed scaling relationship diverges from the expected relationship under morphological/kinematic/dynamic similarity and moves toward the slope expected under weight support, the parameter Δa = (a_allo - a_sim) increases. Implicitly, the authors assume that the larger the Δa, the more the trait is adapted to meet the requirement of weight support. However, Δa will increase as the slope of the allometry moves away from that expected under similarity, regardless of whether it moves toward the weight-support slope (as seen in Fig. 4G). This should be accounted for. Relatedly, in Figure 4H, C_v has a negative %, which indicates that the slope of the observed allometry is less than expected under dynamic similarity, but it is not necessarily true that it is also moving away from the slope for weight support, as implied by the y-axis (although in this case, by happenstance, it is). Further, by focusing solely on the slope, Equation (11) ignores the intercept; one can imagine a scenario where the slope remains the same but the intercept changes, bringing the observed scaling relationship closer to the expected weight-support relationship. In short, there are hidden assumptions in Equation (11) that should be made explicit.

5.Figure 5A. The authors should be explicit about which flies rely on acoustic signals for mate localization and which do not, and these should be indicated on the plot. Also, I think the lines may be mislabeled: the dot-dash line is labeled "low frequency fliers" but corresponds to species with higher wingbeat frequencies. It is also not clear how these lines were calculated. They are not fitted lines, but are instead derived from the model. In that case, how were the intercepts for the two groups calculated? The same issue applies to Figures 7A and 7B. In the caption for Figure 7, the authors state: "Trend lines are based on scaling laws (see Fig. 5) and a statistical fit." How was this statistical fit achieved?

6.In general, the authors overstate the causal implications of their results. While the data are consistent with aerodynamic constraints in general and weight support specifically, and with potential trade-offs involving acoustic production, they are essentially correlational. Future analyses that incorporate phylogenetically independent contrasts would be helpful in establishing causality, but are outside the scope of the current paper.

7.L. 559 and L188. The authors measured body length and width. A little more detail here: length is presumably tip of head to the terminila? But how is thoax width measured? On what plane? An image showing example measuerments as a supplemental figure would be very helpful.

8.A minor point, but the terminology surrounding scaling, allometry, isometry, and similarity is used somewhat inconsistently. For example, isometry has a very specific meaning (literally no change in proportion), yet here it is used more liberally to mean scaling that conforms to expectations under geometric similarity (Table 1). The term similarity is also used in a way that sometimes obscures meaning. Although it is defined at L456, the authors switch between morphological similarity and isometric scaling (L460), giving the impression that similarity equates to isometry, which is not always the case. Perhaps "proportionate" versus "disproportionate" scaling would be clearer. This is admittedly a bit of a minefield, but the authors should think carefully about vocabulary to maximize clarity.

Reviewer #4:

This is a short review, necessarily so because of time constraints, but also because the paper is very good and requires little comment

This is a very impressive work, collecting the specimens, the morphological analysis, flight and kinematics analysis, CFD - the authors have really pushed the limits. The paper is well written, dense, but accessible. The results are presented clearly. If nothing else, the collection of data and the scaling is fascinating. I particularly appreciate the "simple" models that are used to tie the quantitative and descriptive components of the analysis together.

I don't have anything major to object to but do have some more general questions that would be nice to address:

Based on these results, can the authors make any predictions regarding the minimum size/weight that might be able to achieve sustained flight? As scale continues to decrease, and Re goes down, drag continues to rise - what is the theoretical limit.... is there a limit of the required (relative) muscle mass? (although the larger species also have very high fractions of muscle mass - Fig 7B)

The outlier continually demonstrated by Culicomorpha has always fascinated me - it is the cousin that no one can believe they are related to! - Is there anything more that can be said about the pressures that might have led to this evolutionary line? There is a passing comment in the final lines of the paper, but is echoed in the abstract, that reproductive success is here more important than aerodynamic efficiency - what does this refer to?

COMMENTS FROM THE ACADEMIC EDITOR (lightly edited):

I do agree that the paper is worth publishing. This is because such studies combining a range of fields, from phylogeny and functional morphology to biomechanics and physics are extremely hard to execute and therefore quite rare. They also are important for gaining a more comprehensive picture and bridging fields. The decision to revise substantially in four months is reasonable. The authors should still try as hard as they can to address all comments, including R1's, or rebut if necessary.

I have one request of my own and hope the authors have the data:

When invoking sexual selection to explain a pattern, it is necessary to collect sex-specific data. It would be very useful to see if males and females of mosquitos and crane flies show different patterns of wing beat and acoustic production. The expectation here is that males are the ones chasing females and therefore the one looking for signs that guide them to their location. Often these traits are sexually dimorphic and could manifest through differences in the patterns shown by the sexes. My question to the authors is whether they can trace the sexes back in their data and look for differences to support the claims of sexual selection being involves.

# Author comment: Author response to Decision Letter 2

**DOI:** 10.1371/journal.pbio.3003473.r003

2 Apr 2026

Attachment Submitted filename: responses_to_reviewers_round1.pdf

# Peer review: Decision Letter 2

**DOI:** 10.1371/journal.pbio.3003473.r004

**Authors:** Roland Roberts

7 May 2026

Dear Dr Muijres,

Thank you for your patience while we considered your revised manuscript "Diptera flight diversity is shaped by aerodynamic constraints, scaling, and evolutionary trade-offs" for publication as a Research Article at PLOS Biology. This revised version of your manuscript has been evaluated by the PLOS Biology editors, the Academic Editor, and two of the original reviewers.

Based on the reviews and on our Academic Editor's assessment of your revision, we are likely to accept this manuscript for publication, provided you satisfactorily address the remaining points raised by the reviewers, and the following data and other policy-related requests.

a) Regarding the Title, we wonder whether it would be better to put "Dipteran" as an adjective describing "flight diversity" rather than "Diptera" as the noun for the order of insect?

b) Please attend to the remaining requests from reviewer #1.

c) Please address my Data Policy requests below; specifically, we need you to supply the numerical values underlying Figs 1ABC, 2ABCDEF, 3CDEFGHIJ, 4ABCDEFGH, 5ABCDE, 6ABC, 7ABC, 8AB, S1AB, S2, S3AB, either as a supplementary data file or as a permanent DOI’d deposition. I note that you already have an associated DRYAD deposition (DOI: 10.5061/dryad.gxd25480s), but this currently only contains raw data. Please could you complete this deposition with the data and code underlying the Figures?

d) Please cite the location of the data clearly in all relevant main and supplementary Figure legends, e.g. “The data underlying this Figure can be found in S1 Data” or “The data underlying this Figure can be found in DOI: 10.5061/dryad.gxd25480s"; also update your Data Availability Statement accordingly.

e) Please make any custom code available, either as a supplementary file or as part of your data deposition.

f) Please include the URLs of your funders in the Financial Disclosure statement.

As you address these items, please take this last chance to review your reference list to ensure that it is complete and correct. If you have cited papers that have been retracted, please include the rationale for doing so in the manuscript text, or remove these references and replace them with relevant current references. Any changes to the reference list should be mentioned in the cover letter that accompanies your revised manuscript.

In addition to these revisions, you may need to complete some formatting changes, which you will receive in a follow up email. A member of our team will be in touch with a set of requests shortly. If you do not receive a separate email within a few days, please assume that checks have been completed, and no additional changes are required.

We expect to receive your revised manuscript within two weeks.

To submit your revision, please go to https://www.editorialmanager.com/pbiology/ and log in as an Author. Click the link labelled 'Submissions Needing Revision' to find your submission record. Your revised submission must include the following:

- a cover letter that should detail your responses to any editorial requests, if applicable, and whether changes have been made to the reference list

- a Response to Reviewers file that provides a detailed response to the reviewers' comments (if applicable, if not applicable please do not delete your existing 'Response to Reviewers' file.)

- a track-changes file indicating any changes that you have made to the manuscript.

NOTE: If Supporting Information files are included with your article, note that these are not copyedited and will be published as they are submitted. Please ensure that these files are legible and of high quality (at least 300 dpi) in an easily accessible file format. For this reason, please be aware that any references listed in an SI file will not be indexed. For more information, see our Supporting Information guidelines:

https://journals.plos.org/plosbiology/s/supporting-information

*Published Peer Review History*

Please note that you may have the opportunity to make the peer review history publicly available. The record will include editor decision letters (with reviews) and your responses to reviewer comments. If eligible, we will contact you to opt in or out. Please see here for more details:

https://plos.org/published-peer-review-history/

*Press*

Should you, your institution's press office or the journal office choose to press release your paper, please ensure you have opted out of Early Article Posting on the submission form. We ask that you notify us as soon as possible if you or your institution is planning to press release the article.

*Protocols deposition*

To enhance the reproducibility of your results, we recommend that if applicable you deposit your laboratory protocols in protocols.io, where a protocol can be assigned its own identifier (DOI) such that it can be cited independently in the future. Additionally, PLOS ONE offers an option for publishing peer-reviewed Lab Protocol articles, which describe protocols hosted on protocols.io. Read more information on sharing protocols at https://plos.org/protocols?utm_medium=editorial-email&utm_source=authorletters&utm_campaign=protocols

Please do not hesitate to contact me should you have any questions.

Sincerely,

Roli Roberts

Roland Roberts, PhD

Senior Editor

rroberts@plos.org

PLOS Biology

------------------------------------------------------------------------

DATA POLICY:

You may be aware of the PLOS Data Policy, which requires that all data be made available without restriction: http://journals.plos.org/plosbiology/s/data-availability. For more information, please also see this editorial: http://dx.doi.org/10.1371/journal.pbio.1001797

Note that we do not require all raw data. Rather, we ask that all individual quantitative observations that underlie the data summarized in the figures and results of your paper be made available in one of the following forms:

1) Supplementary files (e.g., excel). Please ensure that all data files are uploaded as 'Supporting Information' and are invariably referred to (in the manuscript, figure legends, and the Description field when uploading your files) using the following format verbatim: S1 Data, S2 Data, etc. Multiple panels of a single or even several figures can be included as multiple sheets in one excel file that is saved using exactly the following convention: S1_Data.xlsx (using an underscore).

2) Deposition in a publicly available repository. Please also provide the accession code or a reviewer link so that we may view your data before publication.

Regardless of the method selected, please ensure that you provide the individual numerical values that underlie the summary data displayed in the following figure panels as they are essential for readers to assess your analysis and to reproduce it: Figs 1ABC, 2ABCDEF, 3CDEFGHIJ, 4ABCDEFGH, 5ABCDE, 6ABC, 7ABC, 8AB, S1AB, S2, S3AB. NOTE: the numerical data provided should include all replicates AND the way in which the plotted mean and errors were derived (it should not present only the mean/average values).

IMPORTANT: Please also ensure that figure legends in your manuscript include information on where the underlying data can be found, and ensure your supplemental data file/s has a legend.

Please ensure that your Data Statement in the submission system accurately describes where your data can be found.

------------------------------------------------------------------------

CODE POLICY

Per journal policy, if you have generated any custom code during the course of this investigation, please make it available without restrictions. Please ensure that the code is sufficiently well documented and reusable, and that your Data Statement in the Editorial Manager submission system accurately describes where your code can be found. More information on our Code Policy, what and how to share can be found here: https://journals.plos.org/plosbiology/s/code-availability

Please note that we cannot accept sole deposition of code in GitHub, as this could be changed after publication. However, you can archive this version of your publicly available GitHub code to Zenodo. Once you do this, it will generate a DOI number, which you will need to provide in the Data Accessibility Statement (you are welcome to also provide the GitHub access information). See the process for doing this here: https://docs.github.com/en/repositories/archiving-a-github-repository/referencing-and-citing-content

------------------------------------------------------------------------

DATA NOT SHOWN?

- Please note that per journal policy, we do not allow the mention of "data not shown", "personal communication", "manuscript in preparation" or other references to data that is not publicly available or contained within this manuscript. Please either remove mention of these data or provide figures presenting the results and the data underlying the figure(s).

------------------------------------------------------------------------

REVIEWERS' COMMENTS:

Reviewer #1:

This remains an impressive body of work. Any quibbles I have with language, framing or detail in the main body are unimportant as the description of process is thorough, and readers will have the opportunity to reanalyse/ reframe in their own interest. I strongly appreciate the efforts to make the data usefully available.

One main issue remains of concern – largely concerning the framing/hypotheses.

Constraint cannot be inferred.

Abstract: “Combining these data with our scaling analyses suggests that tiny Diptera are primarily constrained by aerodynamic force production, and maintain weight support through relatively larger wings and increased wingbeat frequencies. In contrast, as Diptera increase in size, hovering flight becomes progressively constrained by power availability, resulting in markedly elevated relative flight-muscle mass among the largest species.”

Disagree. No constraint can be inferred. Aerodynamic force requires power and area. Sufficient power and area is observed – the flies manage to hover. If you took a little bit of the area away from either case (and left all else unchanged), weight would not be supported: force is limiting. If you took a little bit of the power away (and left all else unchanged), weight would not be supported: power is limiting. In both cases. If you found that one of them had a high power margin (so could power an acceleration upwards) they would also have a high lift margin (so could provide the force needed to accelerate upwards).

That small flies have ‘relatively’ large wings, and big flies have ‘relatively’ big muscles is not untrue or uninteresting, but may simply reflect issues with how ‘relatively’ is defined.

Minor points.

I get a bit confused about assumptions relating to geometric, kinematic and dynamic similarity.

I know what geometric similarity is: lengths scale with L, areas with L^2, volumes with L^3, second moments of area (relevant for lift) L^4 and third moments of area (relevant for power) L^5. Kinematic similarity in the context here presumably relates to consistent angles but does not have a time element to it (this could be clarified – kinematics sometimes covers velocities, accelerations too). The dynamic similarity being discussed in this paper relates only to Reynolds number (dynamic similarity of fluids); it may be worth highlighting that this is not the same as would be expected from wave- or terrestrial motions (e.g. Froude number), which relates to the time scaling required to achieve kinematic similarity (e.g. due to ballistic paths in running gaits). If that definition was applied, dynamic similarity would be achieved whenever the centre of mass was supported in steady hover.

Check throughout: should Dipteran be dipteran (unless beginning sentence) – in my training it would have been ‘dipterans are Diptera.

“Insects are the most species-rich group of animals”

Untrue (in detail): arthropods are surely an even more species-rich group of animals.

L51 (Back to the kinematics query)

“One important effect is that, under geometric similarity, the ability of flapping wings to generate sufficient aerodynamic force for weight support decreases with decreasing body size. Tiny insects need to compensate for this through allometric adjustments in wing…”

Disagree. Under geometric similarity mg= constant.L^4 f^2 (see ref [20] etc.). So tiny insects ‘could’ generate sufficient force with just increase in frequency. They do have higher frequencies, but not enough: I agree they do have relatively (relative to mass^1/3) larger wings, and that is interesting – just reframe.

L. 62 “In the inertial regime, drag is relatively low and approximately independent of Reynolds number (D ∝ Re0)”

Untrue. Drag (absolute) scales with the square of length and speed: Re^2. And do you have a citation for that? Drag coefficient certainly scales less strongly than at low Re, but I recall Re^-0.2 being mentioned, probably in Hoerner.

Line 67. Again, drag coefficient is intended.

L. 68. Not drag absolute – coefficient intended. Also probably true for body and waiting to be determined for wings – depends on angle of incidence, and that is a story that comes later.

Check you with to use superscript * to indicate both weight specific and lift coefficient specific.

Reviewer #3:

The authors have done a very thorough job of addressing my concerns with their initial submission. I am happy to recommend publication.

# Author comment: Author response to Decision Letter 3

**DOI:** 10.1371/journal.pbio.3003473.r005

22 May 2026

Attachment Submitted filename: responses_to_reviewers_round_2.pdf

# Editor report: Decision Letter 3

**DOI:** 10.1371/journal.pbio.3003473.r006

**Authors:** Roland Roberts

26 May 2026

Dear Dr Muijres,

Thank you for the submission of your revised Research Article "Dipteran flight diversity is shaped by aerodynamic constraints, scaling, and evolutionary trade-offs" for publication in PLOS Biology. On behalf of my colleagues and the Academic Editor, Abderrahman Khila, I'm pleased to say that we can in principle accept your manuscript for publication, provided you address any remaining formatting and reporting issues. These will be detailed in an email you should receive within 2-3 business days from our colleagues in the journal operations team; no action is required from you until then. Please note that we will not be able to formally accept your manuscript and schedule it for publication until you have completed any requested changes.

Important: I've asked my colleagues to include the following requests alongside their own: Many thanks for updating your Data Availability Statement. However, please could you correct all relevant main and supplementary Figure legends to include the Dryad DOI, e.g. “The data underlying this figure are available at insectflight.eu and in DOI: 10.5061/dryad.gxd25480s"

Please take a minute to log into Editorial Manager at http://www.editorialmanager.com/pbiology/, click the "Update My Information" link at the top of the page, and update your user information to ensure an efficient production process.

PRESS: We frequently collaborate with press offices. If your institution or institutions have a press office, please notify them about your upcoming paper at this point, to enable them to help maximise its impact. If the press office is planning to promote your findings, we would be grateful if they could coordinate with biologypress@plos.org. If you have previously opted in to the early version process, we ask that you notify us immediately of any press plans so that we may opt out on your behalf.

We also ask that you take this opportunity to read our Embargo Policy regarding the discussion, promotion and media coverage of work that is yet to be published by PLOS. As your manuscript is not yet published, it is bound by the conditions of our Embargo Policy. Please be aware that this policy is in place both to ensure that any press coverage of your article is fully substantiated and to provide a direct link between such coverage and the published work. For full details of our Embargo Policy, please visit http://www.plos.org/about/media-inquiries/embargo-policy/.

Thank you again for choosing PLOS Biology for publication and supporting Open Access publishing. We look forward to publishing your study.

Sincerely,

Roli Roberts

Roland G Roberts, PhD, PhD

Senior Editor

PLOS Biology

rroberts@plos.org
